# Bisecting GlcNAc modification diminishes the pro‐metastatic functions of small extracellular vesicles from breast cancer cells

**DOI:** 10.1002/jev2.12005

**Published:** 2020-10-30

**Authors:** Zengqi Tan, Lin Cao, Yurong Wu, Bowen Wang, Zhihui Song, Juhong Yang, Lanming Cheng, Xiaomin Yang, Xiaoman Zhou, Zhijun Dai, Xiang Li, Feng Guan

**Affiliations:** ^1^ Joint International Research Laboratory of Glycobiology and Medicinal Chemistry College of Life Science Northwest University Xi'an P.R. China; ^2^ Department of Breast Surgery The First Affiliated Hospital of Xi'an Jiaotong University Xi'an P.R. China; ^3^ Department of Breast Surgery Tumor Hospital of Shaanxi Province Xi'an P.R. China; ^4^ Department of Breast Surgery The First Affiliated Hospital College of Medicine Zhejiang University Hangzhou P.R. China; ^5^ Department of Oncology The Second Affiliated Hospital of Xi'an Jiaotong Xi'an P.R. China; ^6^ School of Medicine Northwest University Xi'an P.R. China

**Keywords:** bisecting GlcNAc, breast cancer, integrin, MGAT3, small extracellular vesicles

## Abstract

Small extracellular vesicles (sEVs) are enriched in glycoconjugates and display specific glycosignatures. Aberrant expression of surface glycoconjugates is closely correlated with cancer progression and metastasis. The essential functions of glycoconjugates in sEVs are poorly understood. In this study, we observed significantly reduced levels of bisecting GlcNAc in breast cancer. Introduction of bisecting GlcNAc into breast cancer cells altered the bisecting GlcNAc status on sEVs, and sEVs with diverse bisecting GlcNAc showed differing functions on recipient cells. Carcinogenesis and metastasis of recipient cells were enhanced by sEVs with low bisecting GlcNAc, and the pro‐metastatic functions of sEVs was diminished by high bisecting GlcNAc modification. We further identified vesicular integrin β1 as a target protein bearing bisecting GlcNAc. Metastasis of recipient cells was strongly suppressed by high bisecting GlcNAc levels on vesicular β1. Our findings demonstrate the important roles of glycoconjugates on sEVs. Modification of sEV glycosylation may contribute to development of novel targets in breast cancer therapy.

## 1 INTRODUCTION

Small extracellular vesicles (sEVs), a type of nanoparticle (diameter 30–100 nm) present in various body fluids, originate from the endosomal pathway and are secreted by exocytosis into surrounding extracellular space (Raposo & Stoorvogel, 2013). Tumour cell‐derived sEVs can promote angiogenesis (Rak, 2010) and metastasis (Hood, San, & Wickline, 2011), exert immunomodulatory effects (Wolfers et al., 2001), and remodel surrounding parenchymal tissue (Peinado, Lavotshkin, & Lyden, 2011); these processes jointly facilitate tumour progression. sEVs contain proteins, messenger RNAs, microRNAs, long non‐coding RNAs (lncRNAs), and lipids, and mediate various types of cell‐to‐cell communication (Qu et al., 2016; Valadi et al., 2007).

sEV surfaces are covered abundantly by glycoconjugates (proteoglycans, glycoproteins) (Liang et al., 2014; Melo et al., 2015) which are involved in a variety of biological processes, including cell growth, migration, differentiation, tumour invasion, host‐pathogen interactions, and transmembrane signalling (Zhao et al., 2008). Glycoconjugates also play essential roles in vesicular protein sorting and sEV‐cell interactions (Christianson et al., 2013; Liang et al., 2014). Heparan sulfate molecules on recipient cells function as internalizing receptors of tumour cell‐derived sEVs, and are involved in sEV‐mediated signalling activation and triggering of cancer cell migration (Christianson et al., 2013). Lectin/ glycan interactions are associated with recognition and uptake of sEVs by dendritic cells (Dusoswa et al., 2019) and macrophages (Barrès et al., 2010). However, functional roles of glycoconjugates (in comparison with those of proteins and nucleic acids) in sEVs are poorly understood.

A specific type of N‐glycosylation termed bisecting GlcNAc (β1,4‐linked GlcNAc attached to core β‐mannose residue (Figure S1A), catalysed by N‐acetylglucosaminyltransferase MGAT3) plays a regulatory role in processing and elongation of N‐glycans on proteins (Nakano et al., 2019). Relative to controls, mice that lack MGAT3 and bisecting GlcNAc display more rapid development of PyMT‐induced mammary tumours, greater tumour burden, and higher incidence of early metastasis to lung (Song, Aglipay, Bernstein, Goswami, & Stanley, 2010). Bisecting GlcNAc structures on target proteins such as EGFR and integrins play essential roles in cell adhesion and metastasis (Gu & Taniguchi, 2008, Isaji et al., 2004). No studies to date have addressed regulatory effects of bisecting GlcNAc on sEVs. We describe here the manner in which bisecting GlcNAc modulates function of sEVs and physiology of recipient cells.

## 2 MATERIALS AND METHODS

### 2.1 Cell lines and cell culture

Human mammary epithelial cell line (MCF10A) and human mammary carcinoma cell lines (MCF7, SK‐BR‐3, and MDA‐MB‐231 [abbreviated as MDA‐231]) were from the Cell Bank at the Chinese Academy of Sciences (Shanghai, China). MCF7, SK‐BR‐3, and MDA‐231 cells were grown in DMEM. MCF10A cells were grown in DMEM/F12 supplemented with 100 ng/ml cholera enterotoxin, 10 μg/ml insulin, 0.5 μg/ml hydrocortisol, 20 ng/ml EGF, 5% horse serum, 100 UI/ml penicillin, and 100 μg/ml streptomycin at 37°C in 5% CO_2_ atmosphere.

### 2.2 Stable transfection of MGAT3

MGAT3 was amplified via PCR and linked to lentiviral overexpression vector pLVX‐AcGFP‐N1 (Takara; Shiga, Japan). Lentiviral vectors were packed in HEK293T using Lipofectamine 2000 reagent (Thermo Fisher Scientific; San Jose, CA, USA), together with pMD2.G and psPAX2 (Addgene; Cambridge, MA, USA). Lentivirus particles were harvested 48 h after transfection, and MDA‐231 cells were infected with the resultant lentivirus. Transfected cells were cultured in complete medium with puromycin (2 μg/ml) for 4 days. Stable transfectants were selected, and confirmed by western blotting analysis.

### 2.3 Patient samples

Plasma and tissues of normal subjects and BC patients were obtained from the First Affiliated Hospital of Xi'an Jiaotong University. Written informed consent was obtained from all patients in accordance with the Declaration of Helsinki. Experiments using human tissues were approved by the Research Ethics Committee of Northwest University.

### 2.4 Total protein extraction

Cells (∼1 × 10^7^) were detached with trypsin, washed twice with ice‐cold 1 × PBS (0.01 mol/l phosphate buffer containing 0.15 mol/l NaCl, pH 7.4), and added with appropriate amount of RIPA buffer (50 mM Tris, pH 7.2, 1% Triton X‐100, 0.5% sodium deoxycholate, 0.1% SDS, 150 mM NaCl, 10 mM MgCl_2_, 5% glycerol) containing protease inhibitor. Breast cancer (BC) tissues were homogenized using a Dounce homogenizer with 500 μl T‐PER Tissue Protein Extraction Reagent (Thermo Fisher) containing protease inhibitor. Lysate was centrifuged, and supernatant was collected. Protein content was determined by BCA assay (Beyotime Institute of Biotechnology; Haimen, China).

### 2.5 Western blotting analysis

Proteins were loaded and separated by SDS‐PAGE, and gels were transferred onto polyvinylidene difluoride (PVDF) membranes (Bio‐Rad; Hercules, CA, USA). Membranes were blocked with 5% (w/v) BSA in TBST for 1 h at 37°C, probed with primary antibodies overnight at 4°C, and incubated with appropriate HRP‐conjugated secondary antibody. Bands were visualized by enhanced chemiluminescence (ECL; Vazyme Biotech; Nanjing, China).

### 2.6 Immunoprecipitation

Cells were cultured and lysed as described above. Lysates (500 μg) were incubated with 2 μg primary antibody for 2 h at 4°C, and added with 20 μl Protein A/G Plus‐Agarose. The mixture was incubated with rotation overnight at 4°C, rinsed with 1 × PBS, denatured with SDS sample loading buffer, and analysed by western blotting.

### 2.7 Enzyme‐linked immunosorbent assay (ELISA)

96‐well ELISA plates (Jet Biofil; Guangzhou, Guangdong, China) were coated with patient plasma samples, diluted 1:50 in PBS, incubated with shaking (200 rpm) for 2 h at 37°C, blocked with 3% BSA in PBS for 2 h at room temperature (RT), and washed with TPBS. Biotinylated PHA‐E (Vector Laboratories; Burlingame, CA, USA) (2 μg/ml) was added, incubated with shaking (200 rpm) for 2 h at 37°C, and washed. VECTASTAIN ABC reagent (Vector Labs) was added to each well, incubated for 30 min at RT, and washed. TMB substrate kit (Beyotime) was added, acid stop solution (2 M sulfuric acid) was added, and optical density at 450 nm was determined by plate reader.

### 2.8 Lectin histochemistry

Cells were cultured in 24‐well plates containing sterilized coverslips, washed with cold PBS, immobilized with 2% fresh paraformaldehyde for 15 min at RT, permeabilized with 0.2% Triton X‐100 in 1 × PBS for 10 min at RT, blocked with 5% BSA in 1 × PBS overnight at 4°C, and incubated with fluorescence‐labelled lectin (1:500; Vector Labs; Burlingame, CA, USA) overnight at 4°C. Coverslips were stained with DAPI for 10 min at RT, and rinsed with 1 × PBS. Images were captured by fluorescence microscopy (Eclipse E600; Nikon; Tokyo, Japan).

### 2.9 Tissue microarray analysis/ immunohistochemistry

BC tissue microarrays (TMAs) containing 30 cases of BC and para‐carcinoma tissues were from Shanghai Outdo Biotech Co. TMAs or paraffin‐embedded slices of BC tissues were incubated for 1 h at 63°C, deparaffinized in xylene and graded concentrations of alcohol, blocked with 5% BSA in 1 × PBS for 1 h at 37°C, incubated with biotinylated‐lectin or primary antibody overnight at 4°C, rinsed with 1 × PBS, incubated with HRP‐streptavidin or secondary antibody for 1 h at 37°C, visualized with DAB reagent, stained with haematoxylin, and photographed. The mean optical density of the staining signal in breast ducts or cancer nests of TMA were calculated using ImagePro Plus software (Media Cybernetics; Silver Spring, MD, USA).

### 2.10 Identification of proteins with bisecting N‐glycans

Proteins (each sample 1 mg) were concentrated and desalted using size‐exclusion spin ultrafiltration unit (10 KD; Millipore), denatured with 8 M urea, 10 mM DTT, 20 mM IAM (Sigma‐ Aldrich), digested with lysyl endopeptidase (Wako Pure Chemical; Osaka, Japan) at 1:100 (w/w) for 4 h at 37°C as first digestion step, and then digested with trypsin (Promega; Madison, WI, USA) at 1:100 (w/w) overnight at 37°C. Peptides were collected by centrifugation and purified using Oasis HLB cartridges (Waters; Milford, MA, USA). Eluates were lyophilized, dissolved with binding buffer (50 mM NH_4_HCO_3_, 150 mM NaCl, 1 mM CaCl_2_, 1 mM MnCl_2_, pH 7.4), and incubated with 50 μl PHA‐E‐agarose (Vector Labs) overnight at 4°C. The mixture was rinsed with 1 × PBS, and peptides were released by boiling for 10 min. Glycopeptides with bisecting GlcNAc structures were collected by centrifugation, and purified using Oasis HLB cartridges. Two‐dimensional LC‐MS and data analysis were performed using LTQ Orbitrap MS (Thermo Fisher), Byonic software program (Protein Metrics; San Carlos, CA, USA), and MaxQuant software program as described previously (Cox & Mann, 2008; Washburn, Wolters, & Yates, 2001).

### 2.11 Transwell assay

Transwell assay was performed using cell culture inserts (pore size 8 μm; Corning) as per manufacturer's instructions. 2 × 10^4^ cells in serum‐free medium were starved for 24 h, inoculated in upper chamber, and complete medium was added to bottom chamber. After 24 h culture, cells migrated across the membrane were stained with 0.1% crystal violet, and photographed under microscope (magnification 100 ×).

### 2.12 Scratch wound assay

Scratch wound assay was performed as described previously (Tan, Wang, Li, & Guan, 2018). In brief, confluent monolayers of MDA‐MB‐231 cells in 6‐well plate, pretreated with 12 μM mitomycin C for 2 h, were scratched with a pipette tip. Cells were rinsed with PBS, added with DMEM medium. The progress of cell migration into the wound was photographed. Wound tracks were marked, and relative migration distance was calculated using ImagePro Plus software.

### 2.13 Co‐culture of BC cells with transwell inserts

MCF7 cells were co‐cultured with MDA/vec or MDA/MGAT3 cells as described previously (Le et al., 2014). In brief, MCF7 were plated in 0.4‐μm porous transwell inserts (Corning; Cambridge, MA, USA), MDA/vec or MDA/MGAT3 were plated in bottom chamber at ratio 4:1 relative to MCF7, and cells were co‐cultured for three days.

### 2.14 Colony formation assay

Colony formation was performed as described previously (Yu et al., 2008). In brief, cells were plated in a 6‐cm dish, grown 1–2 weeks until small colonies were clearly observed, rinsed twice with PBS, fixed with 4% paraformaldehyde, stained with crystal violet solution, and photographed. Crystal violet was dissolved by acetic acid solution (10%), and absorption at 595 nm was measured.

### 2.15 Cell apoptosis

Cell apoptosis was determined as per manufacturer's instructions. In brief, cells (2 × 10^5^) were incubated with 5 μl FITC‐conjugated annexin V (BioLegend; San Diego, CA, USA) in binding buffer for 10 min, rinsed with PBS, resuspended in binding buffer, and added with 5 μg/ml 7‐AAD (BioLegend). Cells in early apoptosis (annexin V‐positive) and in late apoptosis (annexin V‐positive and 7‐AAD‐positive) were quantified by flow cytometry (ACEA Biosciences; San Diego).

### 2.16 Purification of sEVs by differential ultracentrifugation

sEVs were isolated as described previously (Shurtleff et al., 2017). In brief, cells were cultured in sEV‐free FBS medium for 48 h, and culture supernatants were collected and subjected to successive centrifugations at 300 x *g* for 10 min, 2000 x *g* for 10 min, 10,000 x *g* for 30 min, and 100,000 x *g* for 70 min at 4°C. sEV pellets were rinsed with PBS, collected by ultracentrifugation at 100,000 x *g* for 70 min (Optima XE‐100 ultracentrifuge; Beckman Coulter Life Sciences; Indianapolis, IN, USA), resuspended in 100 μl PBS, and stored at ‐80°C. For carboxyfluorescein succinimidyl ester (CFSE) labelling, sEVs were stained with 10 μM CFSE (Sigma‐Aldrich) for 30 min at 37°C and collected by ultracentrifugation. For purification of sEVs from BC patient plasma, plasma was subjected to successive centrifugations at 2000 x *g* for 30 min, 12,000 x *g* for 45 min, and 110,000 x *g* for 2 h at 4°C. Pellets were resuspended in PBS, filtered (pore size 0.22 μm), collected by ultracentrifugation at 110,000 x *g* for 70 min, and resuspended in 100 μl PBS.

### 2.17 OptiPrep density gradient purification

sEVs were purified by OptiPrep density gradient as described previously (Sung, Ketova, Hoshino, Zijlstra, & Weaver, 2015). In brief, 40%, 20%, 10%, and 5% (w/v) iodixanol solutions were prepared by diluting OptiPrep (60% (w/v) aqueous iodixanol, Axis‐Shield PoC; AS; Oslo, Norway) with 0.25 M sucrose/ 10 mM Tris, pH 7.5 in 14 × 89 mm Ultra‐Clear tubes. sEVs purified by ultracentrifugation were placed on top of the gradient, continuous gradient was established through ultracentrifugation at 100,000 x *g* for 18 h using a Beckman Coulter Optima XE‐100 and Ti45 rotor, and twelve fractions were collected. Each fraction was diluted in PBS, pelleted through another round of ultracentrifugation at 100,000 x *g* for 3 h, and washed with and resuspended in PBS.

### 2.18 Transmission electron microscopy (TEM)

Purified sEVs were applied to carbon‐coated 400 mesh grids (Electron Microscopy Sciences; Fort Washington, PA, USA) for 5 min, washed with PBS, and stained with 2% uranyl acetate for 30 s as described previously (Cianciaruso et al., 2017). Images were obtained by TEM (model H‐7650; Hitachi; Tokyo) at 80 kV.

### 2.19 Immunoelectron microscopy

Immunoelectron microscopy of purified sEVs was performed as described previously (Thery, Amigorena, Raposo, & Clayton, 2006). In brief, carbon‐coated grids were placed on purified sEVs, blocked with 3% BSA, rinsed with PBS, incubated with anti‐CD63 or anti‐β1 primary antibody for 30 min at RT, then incubated with gold‐conjugated secondary antibody for 20 min at RT. sEVs were washed, incubated with 1% glutaraldehyde for 5 min, and stained with 2% uranyl acetate for 30 s. Images were obtained by TEM as described above.

### 2.20 Nanoparticle tracking analysis

sEVs were loaded into a NanoSight LM10 instrument (Malvern; UK), and particles were tracked for 60 s using the NanoSight nanoparticle tracking analysis software program.

### 2.21 Surface biotinylation of sEVs by NHS‐biotin

sEVs were biotinylated with EZ‐Link Sulfo‐NHS‐LC‐Biotin (Thermo Fisher) as described previously (Fraser et al., 2019). In brief, purified sEVs were incubated with 1 mg/ml NHS‐biotin in 300 μl PBS (pH 7.4) for 30 min at RT, and excessive biotinylation reagent was neutralized after labelling by incubating sEVs with 100 mM glycine in PBS for 15 min at RT. Biotinylated sEVs were centrifuged at 100,000 x *g* for 70 min at 4°C to remove the quenching solution, resuspended in PBS, and stored at −80°C.

### 2.22 Sugar treatment

Sugar treatment was performed as described previously (Song et al., 2010). In brief, MCF7 cells were starved for 24 h, incubated with 0.5 M sucrose or lactose in DMEM for 1 h at 37°C, rinsed with PBS, and incubated with sEVs for 2 h at 37°C.

### 2.23 Phosphokinase array assay

MCF7 cells were starved for 16 h and treated with MDA‐231‐ or MDA‐231/MGAT3‐derived sEVs (100 μg/ml) for 1 h. Levels of phosphorylated proteins were determined in cell lysates (200 μg per sample) as per manufacturer's instructions (ARY003B; R&D Systems; Minneapolis, MN, USA). Results were calculated and quantified using Image J software program (NIH). Data were presented as mean intensity relative to a reference spot on the array.

### 2.24 sEV pre‐conditioning of mice

Vec‐sEVs and MGAT3‐sEVs (20 μg in 100 μl PBS) were centrifuged at 4600 x *g* for 1 min at 4°C to remove sedimentable aggregates, then i.v. injected into 6‐ to 8‐week‐old Balb/c nu/nu mice. Two sEV doses were administered per week.

### 2.25 Lung colonization studies

Six‐ to 8‐week‐old female Balb/c nu/nu mice were injected with 2 × 10^6^ cancer cells (MDA/vec, MDA/MGAT3, or MCF7) via tail vein. Bioluminescence was determined 6–8 week after injection. Mice were euthanized 8 weeks after injection, and lungs were fixed, sectioned, and stained with haematoxylin and eosin (H&E) for quantification of metastatic tumour burden.

### 2.26 *In vivo* bioluminescence imaging

Mice were anaesthetized with isoflurane, i.p. administered with 150 μg luciferin (Beyotime) per g body weight, and imaged under using a Photon Imager Optima (IVIS Lumina XRMS series III; PerkinElmer; Waltham, MA, USA). Photon counts in the lung area were analysed using M3 Vision software program (Biospace Lab).

### 2.27 Data analysis

All experiments were reproduced at least three times. All data are represented as mean ± standard deviation (S.D.). Two‐tailed Student's *t*‐test was used for comparison of data sets between two groups, and differences with *P* < 0.05 were considered statistically significant. Statistical analyses were performed using GraphPad Prism V. 7.0 software program. Notations in figures: :, *P* < 0.05; ::, *P*< 0.01; :::, *P*< 0.001.

## 3 RESULTS

### 3.1 Expression of bisecting GlcNAc in breast cancer cells

**FIGURE 1:**
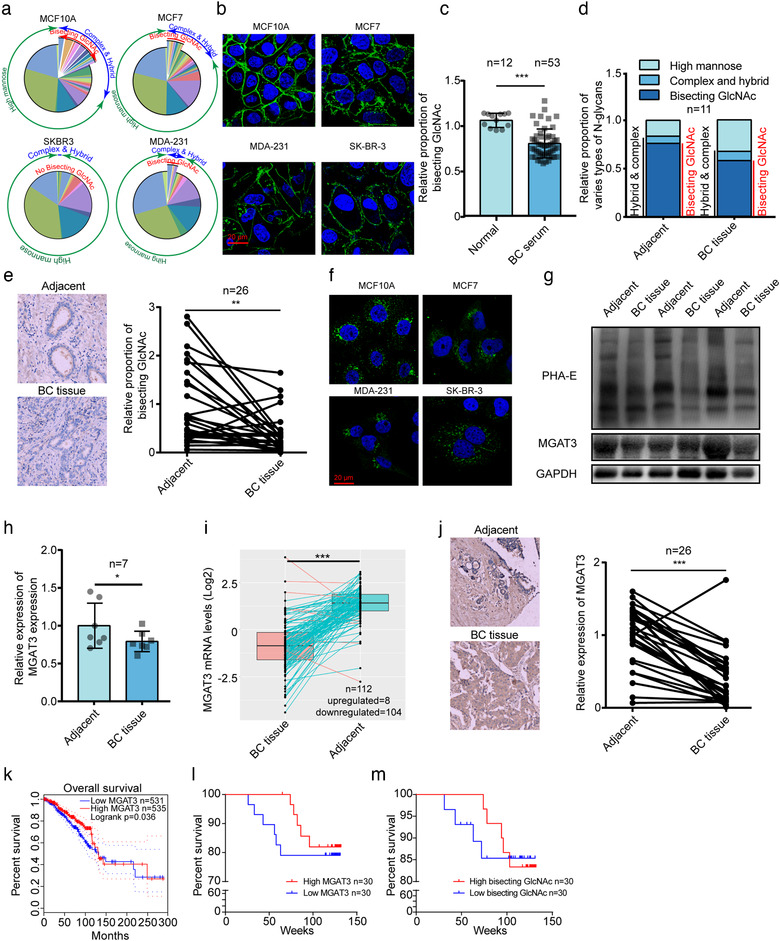
Expression of bisecting GlcNAc in breast cancer cells. (a) Relative proportions of N‐glycans in human breast cells were calculated by dividing intensity of given type of N‐glycan by total intensity of sample. (b) Bisecting GlcNAc in normal mammary epithelial cells and BC cells evaluated by PHA‐E lectin staining. (c) Bisecting GlcNAc in serum of BC patient evaluated by ELISA. (d) MALDI‐TOF‐MS analysis of N‐glycans from adjacent normal and matched BC tissue. N‐Glycans were divided into two groups (complex and hybrid; high‐mannose type). Relative proportion of bisecting GlcNAc in complex and hybrid is shown. (e) Bisecting GlcNAc in BC tissues in TMA. Immunohistochemistry of representative paired clinical tissues is shown. (f) MGAT3 expression in normal mammary epithelial cells and BC cells evaluated by immunofluorescence. (g) Representative image of MGAT3 expression and bisecting GlcNAc levels in adjacent normal and matched BC tissues. (h) MGAT3 expression in adjacent normal and matched BC tissues, determined by western blotting and Image Pro Plus software program. (i) mRNA expression of MGAT3 in 112 adjacent normal and matched BC tissues in TCGA database. (j) Differential MGAT3 expression of BC tissues in TMA. (k) Overall survival of dichotomized MGAT3 expression in BC patients in TCGA database using GEPIA platform. (l, m) Overall survival of dichotomized MGAT3 expression (l) and bisecting GlcNAc levels (m) in BC patients by TMA

We previously reported decreased levels of bisecting GlcNAc in an epithelial‐mesenchymal transition (EMT) model of BC cells (Tan et al., 2014; Tan et al., 2018). In the present study, we examined bisecting GlcNAc levels in additional BC cells, and in clinical serum and tissue samples. Levels of bisecting GlcNAc were significantly lower in human and mouse BC cells relative to normal epithelial cells by MALDI‐TOF/TOF‐MS analysis (Figure 1a and Supporting Information Figures S1B, C, S2A, B, S3A, B; Tables S1, S2) and by lectin staining with PHA‐E lectin, which specifically recognizes bisecting GlcNAc (Nagae et al., 2014) (Figure 1b). ELISA with PHA‐E revealed significantly lower levels of bisecting GlcNAc in serum of BC patients relative to healthy control subjects (Figure 1c). Significantly lower levels of bisecting GlcNAc in cancer tissues relative to normal tissues were demonstrated by MALDI‐TOF/TOF‐MS analysis (Figure 1d and Supporting Information Figure S4; Table S3), which further confirmed by tissue microarray analysis (TMA) (Figure 1e).

Biosynthesis of bisecting GlcNAc is catalysed exclusively by MGAT3. MGAT3 expression level was reduced in BC cells relative to normal breast epithelial cells (Figures 1f and Supporting Information Figure S5A), and in BC tissues (Figures 1g, h and Supporting Information Figure S5B). Analysis of mRNA expression of BC patients from The Cancer Genome Atlas (TCGA) database revealed significantly lower MGAT3 expression in BC tissues relative to adjacent tissues (Figure 1I), which was further confirmed by TMA (Figure 1j). Similarly, the down‐regulated MGAT3 expression was observed in various BC stages (Figures S5C, S6A‐D), in different subtypes (Figure S6E), and in metastatic BC tissues relative to non‐metastatic BC tissues (Figure S6F). In Kaplan‐Meier curve analysis, overall survival was significantly correlated with MGAT3 expression (Figure 1k). Similarly, TMA showed that high MGAT3 expression and bisecting GlcNAc levels were closely correlated with higher overall survival (Figure 1l, m). These findings indicate that aberrant levels of bisecting N‐glycans in BC are due to downregulation of MGAT3 expression.

### 3.2 Effect of conditional medium from MDA/vec and MDA/MGAT3 on migratory ability of MCF7 cells

**FIGURE 2:**
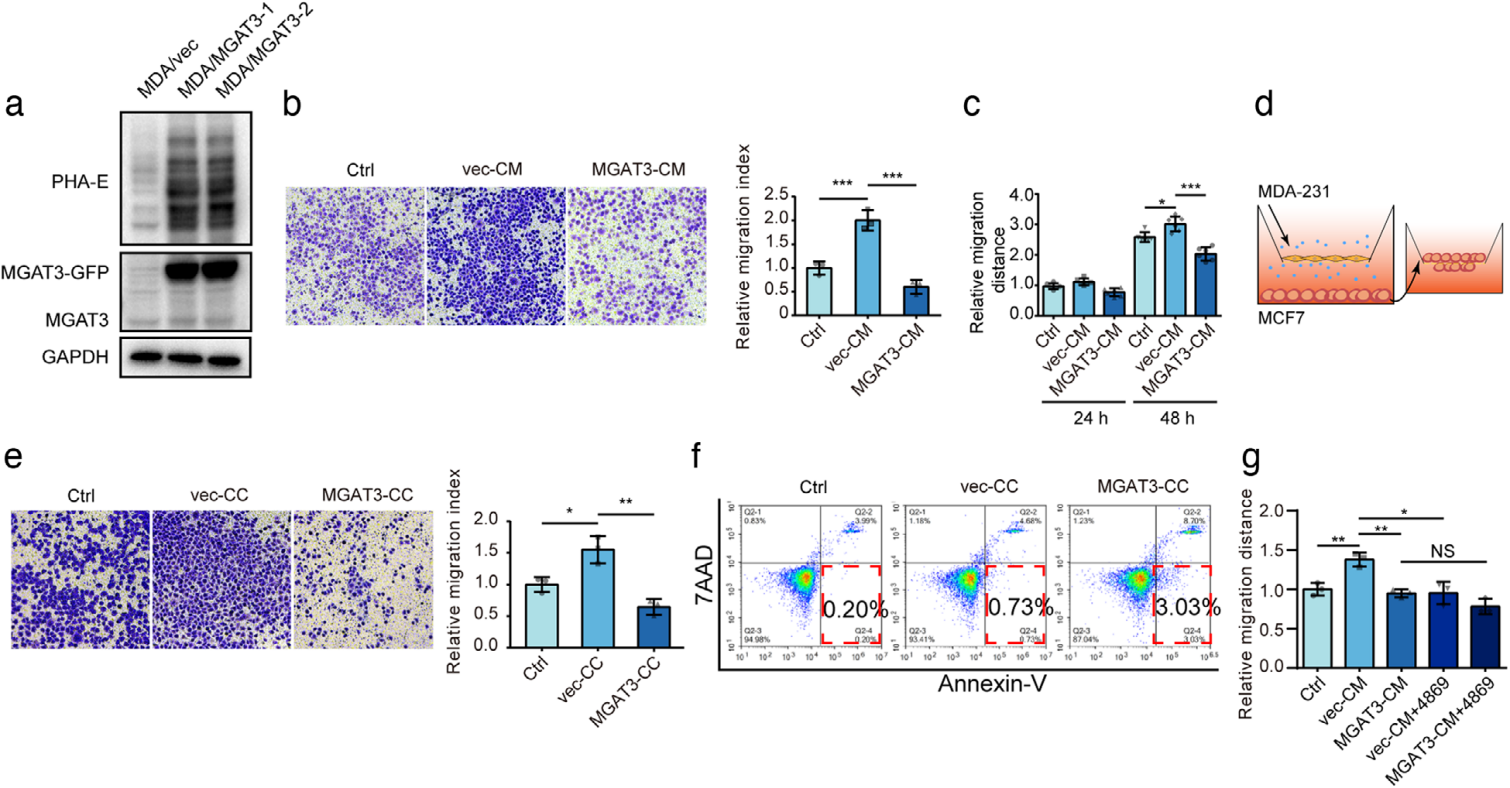
Effects of bisecting GlcNAc modification on conditional medium. (a) MGAT3 expression and bisecting GlcNAc levels in MDA/vec and MDA/MGAT3 cells. (b) Migratory ability of MCF7 treated with vec‐CM or MGAT3‐CM. (c) Scratch wound assay of MCF7 treated with vec‐CM or MGAT3‐CM. (d) Co‐incubation of MCF7 with MDA/vec or MDA/MGAT3 (schematic). (e) Migratory ability of MCF7 co‐cultured with MDA/vec (termed vec‐CC) or MDA/MGAT3 cells (termed MGAT3‐CC). (f) Apoptosis of MCF7 co‐cultured with MDA/vec and MDA/MGAT3. (g) Migratory ability of MCF7 treated with vec‐CM or MGAT3‐CM in presence of GW4869

To evaluate the biological function of bisecting GlcNAc, we introduced MGAT3 into MDA‐MB‐231 cells, and established a stable transfectant (termed MDA/MGAT3) with high level of bisecting GlcNAc (Figure 2a). MDA/MGAT3 cells, relative to MDA‐MB‐231, showed significantly reduced proliferation, clonogenic survival, and cell migration (Figure S7A‐C).

Since the decreased levels of bisecting GlcNAc was detected in serum of BC patients (Figure 1c), we are interested if secreted components from malignant cells could affect the behaviour of recipient cells. We treated low‐metastatic BC cells MCF7 with conditioned medium from vector control and from MDA/MGAT3 cells (termed respectively vec‐CM and MGAT3‐CM). Migratory ability of MCF7 was enhanced by vec‐CM, but inhibited by MGAT3‐CM treatment (Figure 2b, c). Similarly, expression of vimentin (VM) and fibronectin (FN) (mesenchymal markers) was increased in vec‐CM‐treated MCF7 but reduced in MGAT3‐CM‐treated MCF7 (Figure S8A). Experiments using a co‐culture system (Figure 2d) showed that MCF7 migratory ability was enhanced by soluble compounds released by MDA/vec but not by MDA/MGAT3 (Figure 2e). Flow cytometric analysis revealed increased apoptosis in MCF7 co‐incubated with MDA/MGAT3, but not in MCF7 co‐incubated with MDA/vec (Figure 2f). Co‐incubation with MDA/vec or MDA/MGAT3 cells had no notable effect on MCF7 cell proliferation (Figure S8B).

In view of the role of sEVs in mediating cancer progression and metastasis, we treated MDA/vec and MDA/MGAT3 cells with GW4869, a N‐SMase2 inhibitor that blocks ceramide‐mediated release of sEVs (Trajkovic et al., 2008). GW4869 treatment suppressed the migratory effect of vec‐CM on control MCF7 cells (Figure 2g), suggesting that sEVs from MDA/vec and MDA/MGAT3 cells have differing functions on recipient cells.

### 3.3 Effect of bisecting GlcNAc‐bearing sEVs on migratory ability of MCF7 cells

**FIGURE 3:**
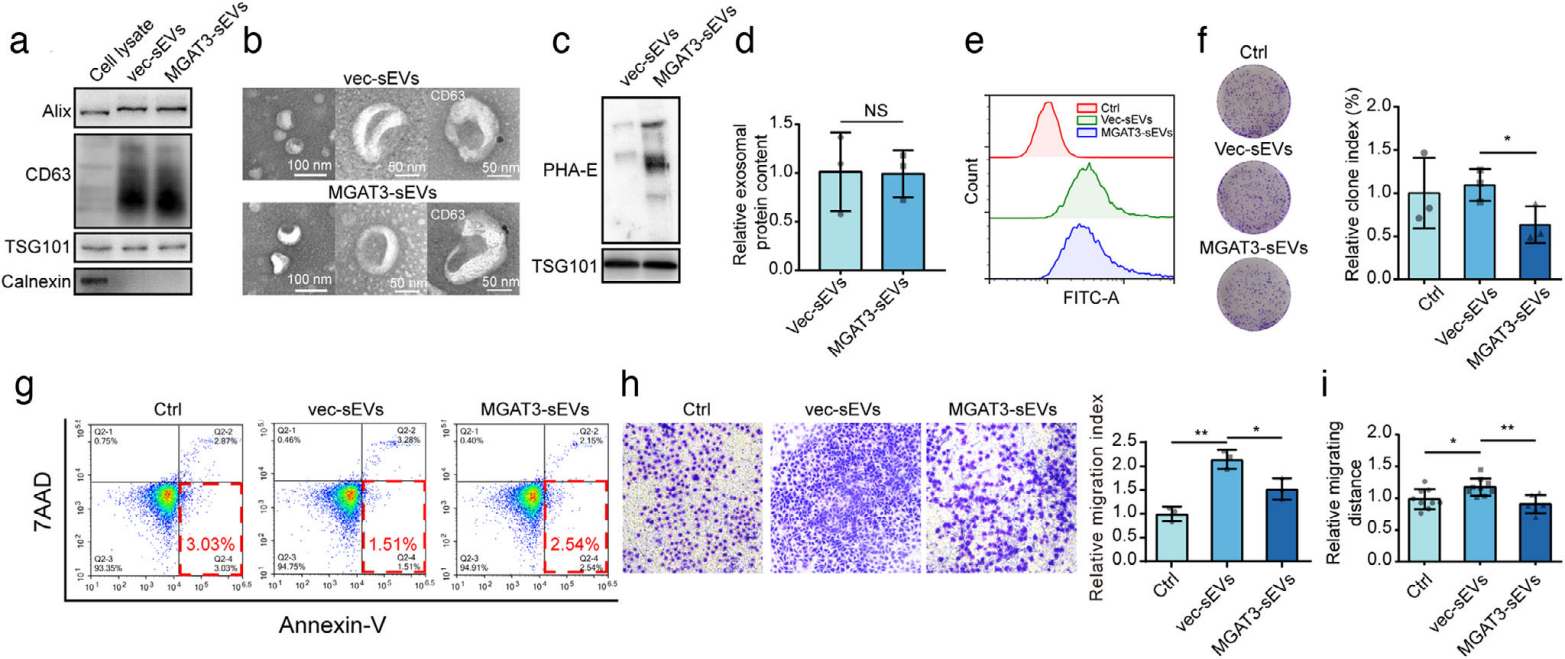
Effects of bisecting GlcNAc modification on sEVs. (a) Expression of sEV marker in vec‐sEVs and MGAT3‐sEVs. (b) Morphology (evaluated by TEM) of sEVs and immunogold‐labelled CD63 on vec‐sEVs and MGAT3‐sEVs. (c) Bisecting GlcNAc levels of vec‐sEVs and MGAT3‐sEVs. (d) sEV secretion in control and MDA‐231/MGAT3. (e) Representative graph of uptake of CFSE‐labelled sEVs in MCF7, by flow cytometry. (f, g) Colony formation ability (f) and apoptosis (g) of MCF7 treated with vec‐sEVs and MGAT3‐sEVs. (h, i) Transwell assay (h) and scratch wound assay (i) of MCF7 treated with vec‐sEVs or MGAT3‐sEVs

We isolated sEVs from MDA/vec and MDA/MGAT3 (respectively termed vec‐sEVs and MGAT3‐sEVs) by a well‐established differential centrifugation method (Shurtleff et al., 2017). They both displayed clear expression of sEV markers (CD63, Alix, TSG101) (Figure 3a), and sphere‐like morphology and diameter (Figures 3b and Supporting Information Figure S9A). Bisecting GlcNAc levels were higher in MGAT3‐sEVs than in vec‐sEVs (Figure 3c). Bisecting GlcNAc modification had no effect on secretion or morphology of sEVs (Figure 3b, d).

sEVs were labelled with carboxyfluorescein diacetate succinimidyl ester (CFSE) and fed to MCF7 cells for measurement of cellular uptake. Vec‐sEVs and MGAT3‐sEVs were both efficiently internalized (Figures 3e and Supporting Information Figure S9B). Colony formation of MCF7 was slightly enhanced by vec‐sEVs, but significantly inhibited by MGAT3‐sEVs treatment (Figure 3f). Compared to control group, apoptosis was clearly decreased in MCF7 cells treated with vec‐sEVs, and slightly decreased in MCF7 cells treated with MGAT3‐sEVs (Figure 3g). Vec‐sEVs and MGAT3‐sEVs did not differ notably in regard to effect on proliferation (Figure S9C). Migratory ability of MCF7 cell was enhanced by vec‐sEVs, but not by MGAT3‐sEVs (Figure 3h, i).

Moreover, we treated other recipient cells, including normal epithelial cell MCF10A, and stromal cells with vec‐sEVs and MGAT3‐sEVs. Vec‐sEVs could stimulate the epithelial‐mesenchymal transition (EMT) of MCF10A cells, and induce cancer‐associated fibroblast‐like properties of stromal cells. However, these pre‐conditioning ability of vec‐sEVs was eliminated by the bisecting GlcNAc modification (Figure S10A‐C). These findings suggest that bisecting GlcNAc structures inhibit carcinogenic effects of sEVs from high‐metastatic cells.

### 3.4 Identification of glycoproteins with bisecting GlcNAc by mass spectrometry analysis

**FIGURE 4:**
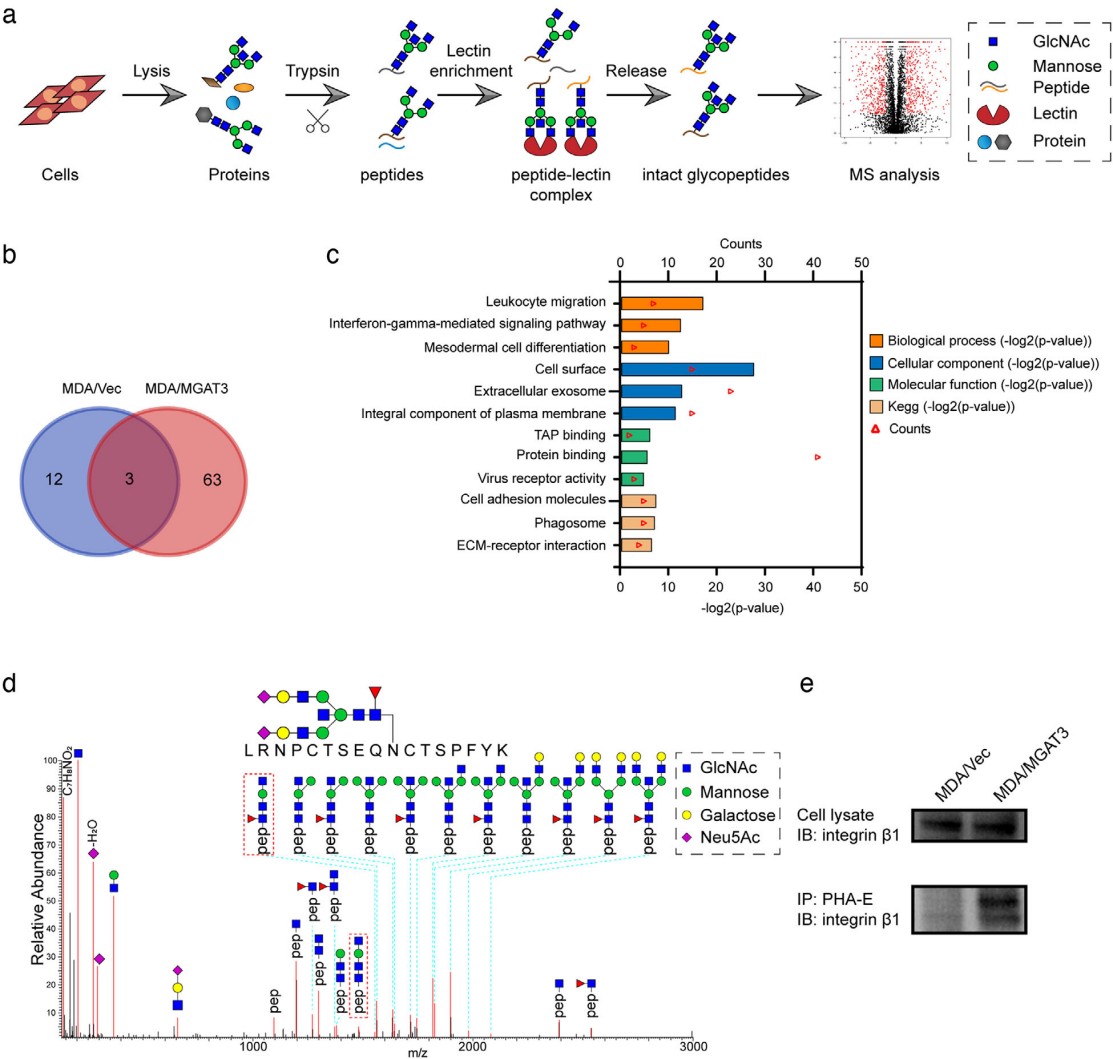
Identification of integrin β1 as the target protein bearing bisecting GlcNAc. (a) Intact glycoproteomic analysis by combination of PHA‐E enrichment and LC‐MS/MS (schematic). (b) Venn diagram of numbers of identified glycoproteins bearing bisecting GlcNAc structures in control and MDA‐231/MGAT3. (c) Gene ontology (GO) classification and KEGG pathway analysis of these glycoproteins. (d) Representative MS/MS spectrum of peptide CHEGN^#^GTFECGACR of β1 with bisecting GlcNAc in MDA/MGAT3 cells. (e) Expression of β1 with bisecting GlcNAc in control and MDA‐231/MGAT3 by immunoprecipitation assay.

As one specific type of N‐glycans, bisecting GlcNAc structures bind to Asn residues of proteins. To identify target glycoproteins having reduced bisecting GlcNAc level in BC cells, we performed intact glycoproteomic analysis by combination of PHA‐E enrichment and triplicate LC‐MS/MS (Figure 4a). Based on identification of [pep+N3H] and [pep+N3HF] as characteristic ions for glycopeptides bearing bisecting GlcNAc (Dang et al., 2019), we identified a total of 78 glycoproteins in MDA/vec and MDA/MGAT3 cells ( Figure 4b; Table S4), and subjected them to gene ontology (GO) enrichment and KEGG pathway analysis. As shown in Figure 4c, the main cellular component categories were cell surface, extracellular exosome, and integral component of plasma membrane. Among the target glycoproteins, integrin β1 (referred to hereafter as “β1” for convenience) recognized bisecting GlcNAc on intact glycopeptides by [pep+N3H] (Figure S11A) and [pep+N3HF] (Figures 4d and Supporting Information Figure S11B) in MDA/MGAT3 cells, but not in MDA/vec cells (Table S4). To validate the mass spectrometry data, immunoprecipitation with PHA‐E was performed. We found that β1 level was the same in MDA/vec and MDA/MGAT3, but level of bisecting GlcNAc on β1 was much higher in MDA/MGAT3 than in MDA/vec (Figure 4e).

### 3.5 Transfer of vesicular β1 bearing bisecting GlcNAc to recipient cells

**FIGURE 5:**
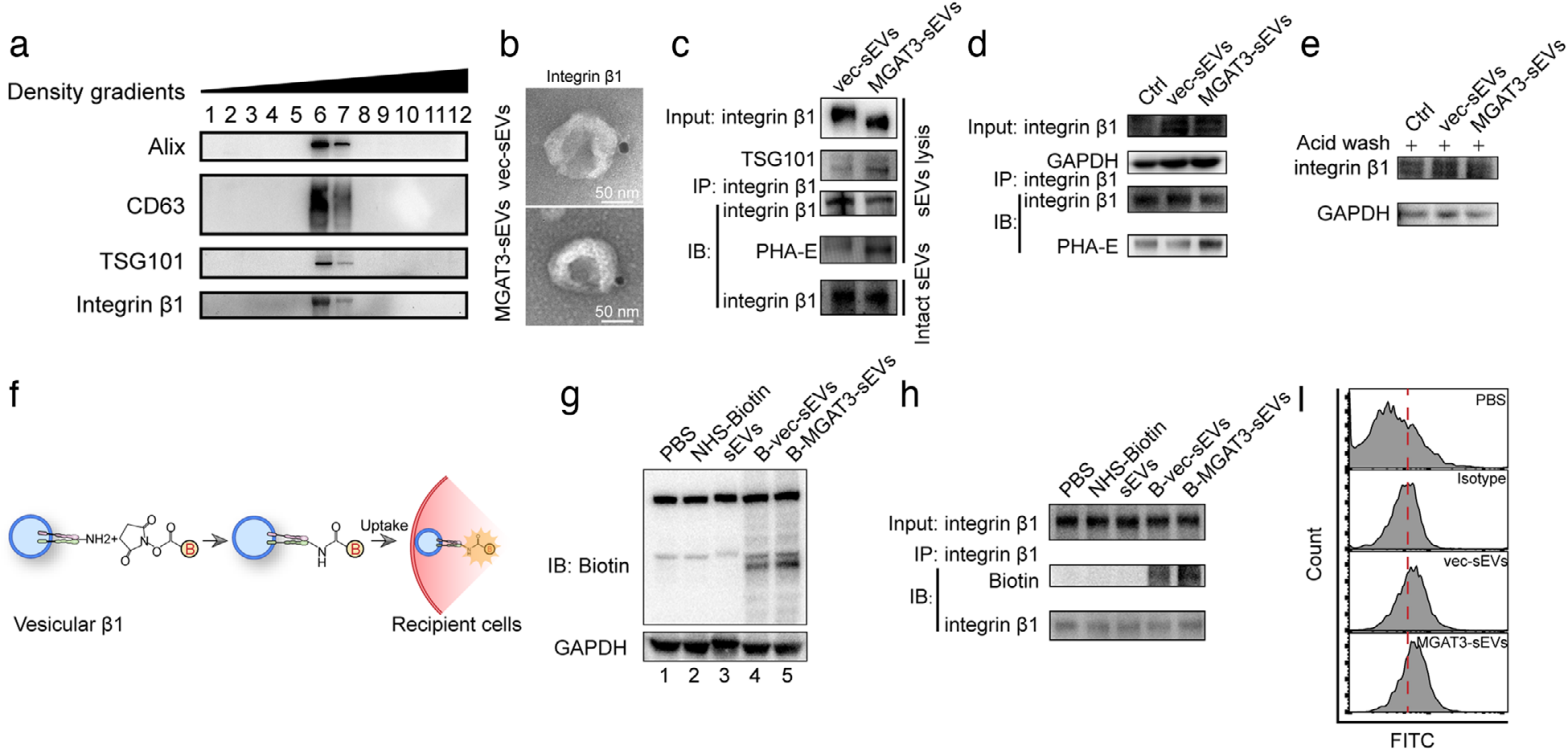
Transfer of vesicular β1 to recipient cells. (a) Western blotting analysis of density gradient fractionation of sEVs from MDA‐231 cells. (b) TEM images of immunogold‐labelled β1 on vec‐sEVs and MGAT3‐sEVs. **(C)** Expression and bisecting GlcNAcylation of β1 from vec‐sEVs and MGAT3‐sEVs evaluated by immunoprecipitation (IP) assay and western blotting. (d) Expression and bisecting GlcNAcylation of β1 on MCF7 treated with vec‐sEVs or MGAT3‐sEVs, evaluated by IP assay and western blotting. (e) Expression of β1 in MCF7 treated with vec‐sEVs and MGAT3‐sEVs after washing with acid wash buffer, analysed by western blotting. (f) Labelling of sEVs with Sulfo‐NHS‐LC‐Biotin (schematic). (g) Sulfo‐NHS‐LC‐Biotin‐labelled sEVs were taken up by MCF7, and Sulfo‐NHS‐LC‐Biotin‐labelled proteins were determined by western blotting. (h) Uptake of Sulfo‐NHS‐LC‐Biotin‐labelled vesicular β1 by MCF7, evaluated by IP assay and western blotting. (i) Expression of β1 on MCF7 plasma membrane after 24 h sEV treatment, evaluated by flow cytometric analysis

Next, expression and glycosylation of β1 on sEVs were evaluated. The presence of β1 on sEVs (termed vesicular β1) was clearly revealed by density gradient fractionation (Figure 5a) and immunoelectron microscopy (Figure 5b). And we ruled out the possibility of load of overexpressed MGAT3 into MGAT3‐sEVs (Figure S12A). β1 level was the same in vec‐sEVs and MGAT3‐sEVs, similar to expression pattern in donor cells, but bisecting GlcNAc level was significantly higher in vesicular β1 of MGAT3‐sEVs (Figure 5c). Vesicular β1 was delivered efficiently to MCF7 cells by both vec‐sEVs and MGAT3‐sEVs (Figure 5d), indicating that sEV endocytosis is not affected by bisecting GlcNAc modification. β1 expression in MCF7 was enhanced following sEV endocytosis, and β1 transferred from MGAT3‐sEVs in MCF7 showed higher levels of bisecting GlcNAc (Figure 5d). Transfer of vesicular β1 expression into MCF7 was confirmed by acid wash, ruling out the possibility that sEVs can be bound externally to recipient cell surface (Figure 5e). In another experiment, vec‐sEVs and MGAT3‐sEVs were labelled with NHS‐biotin (De La Torre‐Escudero et al., 2019) (Figures 5f, S12B). Labelled sEVs were taken up by MCF7 (Figure 5g), and NHS‐biotin‐labelled vesicular β1 was clearly transferred to MCF7 (Figure 5h). Increase of β1 level on MCF7 surface following treatment with vec‐sEVs and MGAT3‐sEVs was confirmed by flow cytometry, indicating that vesicular β1 was endocytosized and rapidly presented on plasma membrane (Figure 5i).

### 3.6 Bisecting GlcNAc suppressed vesicular β1 function

**FIGURE 6:**
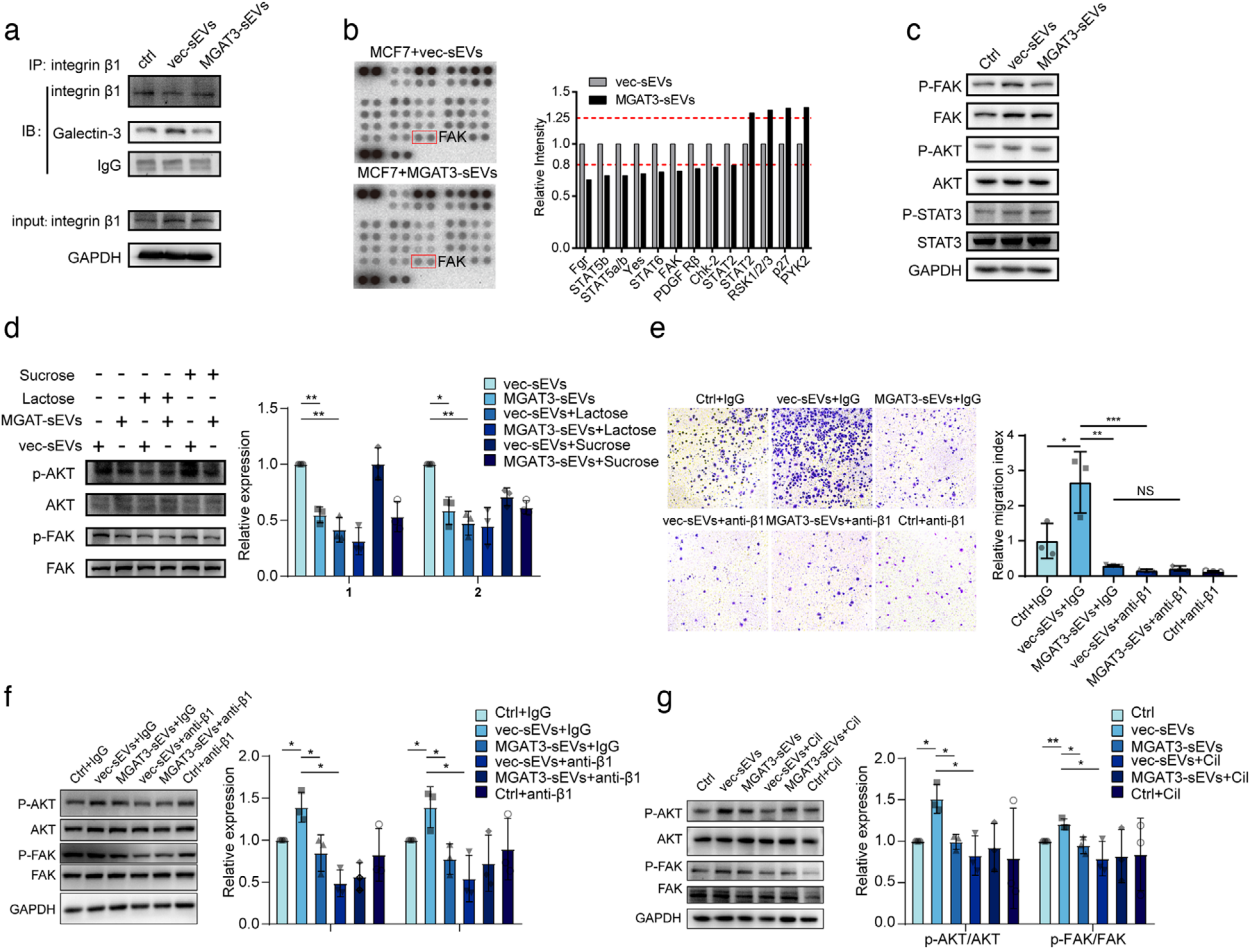
High bisecting GlcNAc level inhibited metastasis of recipient cells via vesicular β1. (a) Galectin‐3/ β1 interaction in MCF7 treated with vec‐sEVs or MGAT3‐sEVs. (b) Levels of phosphorylated kinases in MCF7 treated with vec‐sEVs and MGAT3‐sEVs. (c) Activation of FAK/AKT signaling and suppression of STAT3 signaling in MCF7 treated with vec‐sEVs and MGAT3‐sEVs, analyzed by western blotting. (d) Activation of FAK/AKT signaling in MCF7 treated with vec‐sEVs and MGAT3‐sEVs in the presence of lactose or sucrose. (e) Migratory ability of MCF7 cells treated with vec‐sEVs or MGAT3‐sEVs in the presence of blocking antibody against β1, evaluated by transwell assay. (f, g) Expression of FAK/AKT signaling in MCF7 treated with vec‐sEVs or MGAT3‐sEVs in the presence of anti‐β1 antibody (f) or cilengitide (Cil) (g).

Galectin‐3 has been shown to bind β1 and activate associated signaling (Lakshminarayan et al., 2014), and galectin‐3/ β1 interaction is inhibited by bisecting GlcNAc modification (Kariya, Kawamura, Tabei, & Gu, 2010). Similarly, galectin‐3/ vesicular β1 interaction was notably stronger in vec‐sEV‐treated MCF7 than in MGAT3‐sEV‐treated MCF7 (Figure 6a). These findings suggest that binding of vesicular β1 to galectin‐3 stimulates downstream signaling in recipient cells. Using phospho‐kinase array assays, we found that FAK signaling in MCF7 was activated by vec‐sEVs, but suppressed by MGAT3‐sEVs (Figure 6b, c). Activation of FAK/AKT signalling pathway in MGAT3‐sEV‐treated MCF7 was suppressed by lactose (which competitively binds galectins) but not by sucrose (Figure 6d). Thus, bisecting GlcNAc suppressed galectin‐3/ vesicular β1 interaction, and inactivated FAK/AKT signaling.

As integrin β1 was identified as target protein of bisecting GlcNAc on sEVs, we blocked vesicular β1 with neutralized antibody (P5D2) against β1. Similar as Figure 3h, vec‐sEVs treatment enhanced the migration of MCF7 cells, compared to control group, while the migration of MCF7 could be clearly inhibited by the MGAT3‐sEV, or by the addition of neutralized antibody (Figure 6e). Consistently, blocking of vesicular β1 by antibody or cilengitide (an integrin‐targeting cyclic RGD pentapeptide (Mas‐Moruno, Rechenmacher, & Kessler, 2010)), inactivated FAK/AKT signaling in MCF7 regardless of presence or absence of vec‐sEVs or MGAT3‐sEVs (Figure 6f, g). These data indicated that bisecting GlcNAc modification could suppress vesicular β1 function, similar to the addition of neutralized antibody or cilengitide.

To further evaluate the possible function of bisecting GlcNAc on sEVs, we cleaved the bisecting GlcNAc on sEVs by PNGase F (Figure S13A), and revealed that the interaction between integrin β1 and galectin‐3, FAK‐AKT signaling and metastatic ability of sEVs pre‐treated MCF7 were inhibited by PNGase F treatment (Figure S13B‐D). We further mutated glycosylation sites on the PSI and upstream region of the hybrid domain, and I‐like domain of integrin β1 (containing bisecting GlcNAc glycosylation sites, Figure 4d) in MDA‐231 cells, and revealed that N‐glycans on I‐like domain is essential for the activation of related signalling and migratory ability of both donor and recipient cells (Figure S14A‐F). These results above suggested that N‐glycosylation, especially bisecting GlcNAc, on the sEV surface is able to modulate the function of vesicular integrin β1, and further affect the metastatic ability and related signaling pathway in recipient cells.

### 3.7 Bisecting GlcNAc inhibited pro‐metastatic effects of sEVs

**FIGURE 7:**
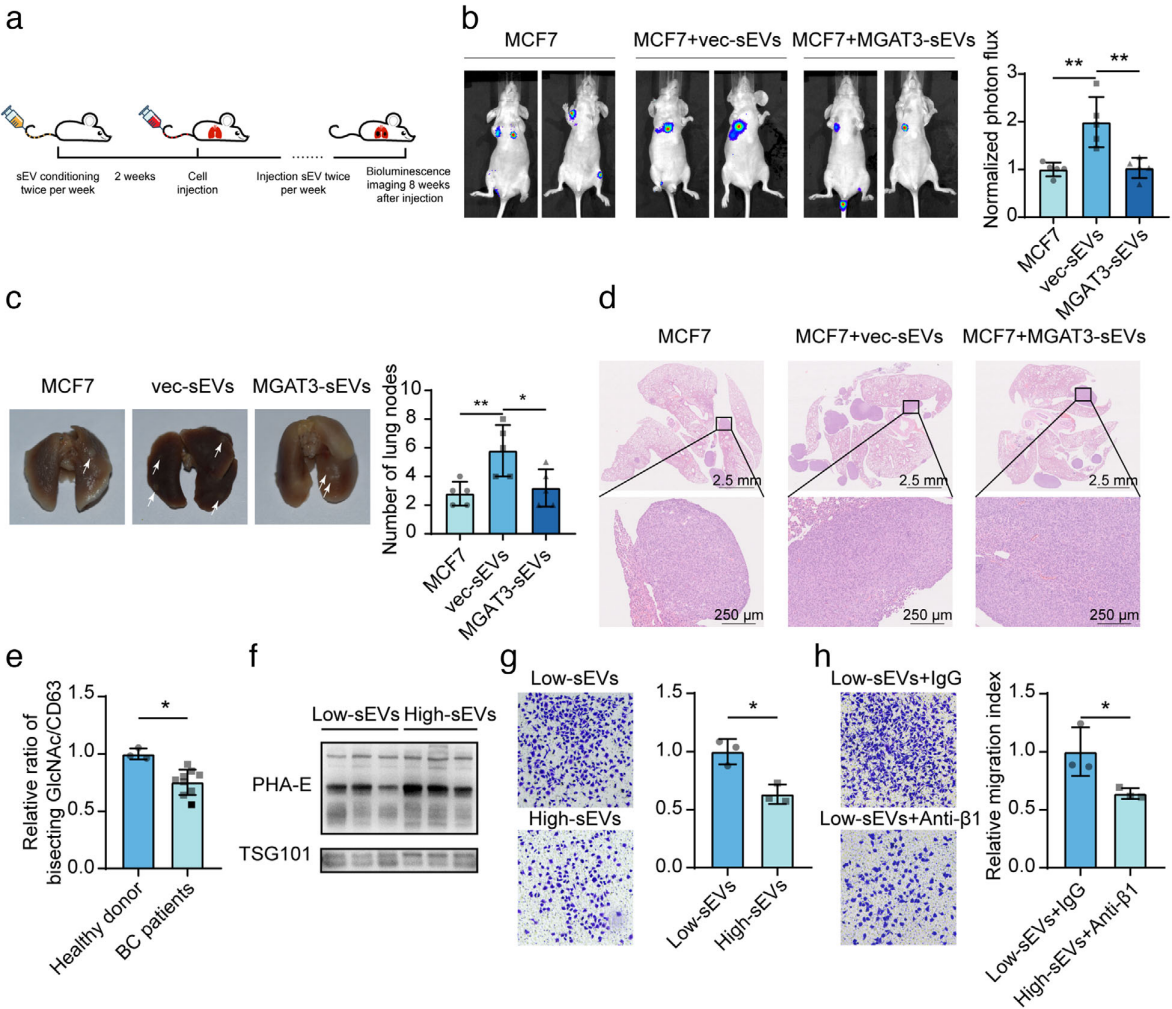
Bisecting GlcNAc inhibited pro‐metastatic effects of sEVs. (a) Experimental setup (schematic). (b) Luciferase activity at week 8 after injection of MCF7 cells pre‐conditioned with PBS, vec‐sEVs or MGAT3‐sEVs (*n* = 5). (c, d) Representative photographs (c) and haematoxylin and eosin (H&E) staining (d) of lungs. (e) Levels of bisecting GlcNAc on sEVs from BC patients and healthy donor. (f) Plasma sEVs from BC patients with low and high levels of bisecting GlcNAc (termed low‐sEVs and high‐sEVs). (g) Migratory ability of MCF7 treated with low‐ and high‐sEVs. (h) Migratory ability of MCF7 treated with low‐sEVs in the presence of anti‐β1 antibody

Next, we examined whether bisecting GlcNAc could inhibit the pro‐metastatic function of sEVs using mouse model. We pre‐conditioned the nude mice with vec‐sEVs or MGAT3‐sEVs, and then i.v. injected with MCF7 cells (Figure 7a). As expected, incidence, numbers and areas of lung metastasis nodules was significantly enhanced by vec‐sEVs but not by MGAT3‐sEVs (Figure 7b‐d). Moreover, immunohistochemistry analysis of lung metastasis nodules revealed the increased expression of two cancer‐associated fibroblast (CAF) markers CD44 and PDGFRβ (Gascard & Tlsty, 2016), in vec‐sEVs pre‐conditioning lung metastasis nodules. And the recruitment and activation of CAF functions of sEVs was diminished by high bisecting GlcNAc modification (Figure S15). Moreover, to validate the function of vesicular integrin β1, we compared the metastasis level of mice injected with vec‐sEVs pre‐incubated with/ without neutralized antibody against integrin β1. Our data demonstrated that the incidence (Figure S16A&B), numbers (Figure S16B) and areas of lung metastasis nodules (Figure S16C) was significantly enhanced by vec‐sEVs, but suppressed by neutralized antibody against integrin β1.

As shown in Figure 1c, bisecting GlcNAc in serum of BC patients significantly lower than healthy donors. We further found lower levels of bisecting GlcNAc on sEVs from BC patients plasma compared to which from healthy donor (Figure 7e). Bisecting GlcNAc levels in sEVs from plasma of BC patients were designated as “high‐sEVs” or “low‐sEVs” on the basis of high vs. low bisecting GlcNAc level (Figure 6f; patient information summarized in Table S5). Migratory ability was greater in low‐sEV‐treated than in high‐sEV‐treated MCF7 (Figure 6g), and low‐sEV‐induced MCF7 migration was significantly inhibited by blocking of β1 with neutralized antibody (Figure 6h). These data confirmed that bisecting GlcNAc modification could suppress vesicular β1 function, and further inhibit the pro‐metastatic effects of sEVs from high‐metastatic BC cells.

## 4 DISSCUSSION

**FIGURE 8:**
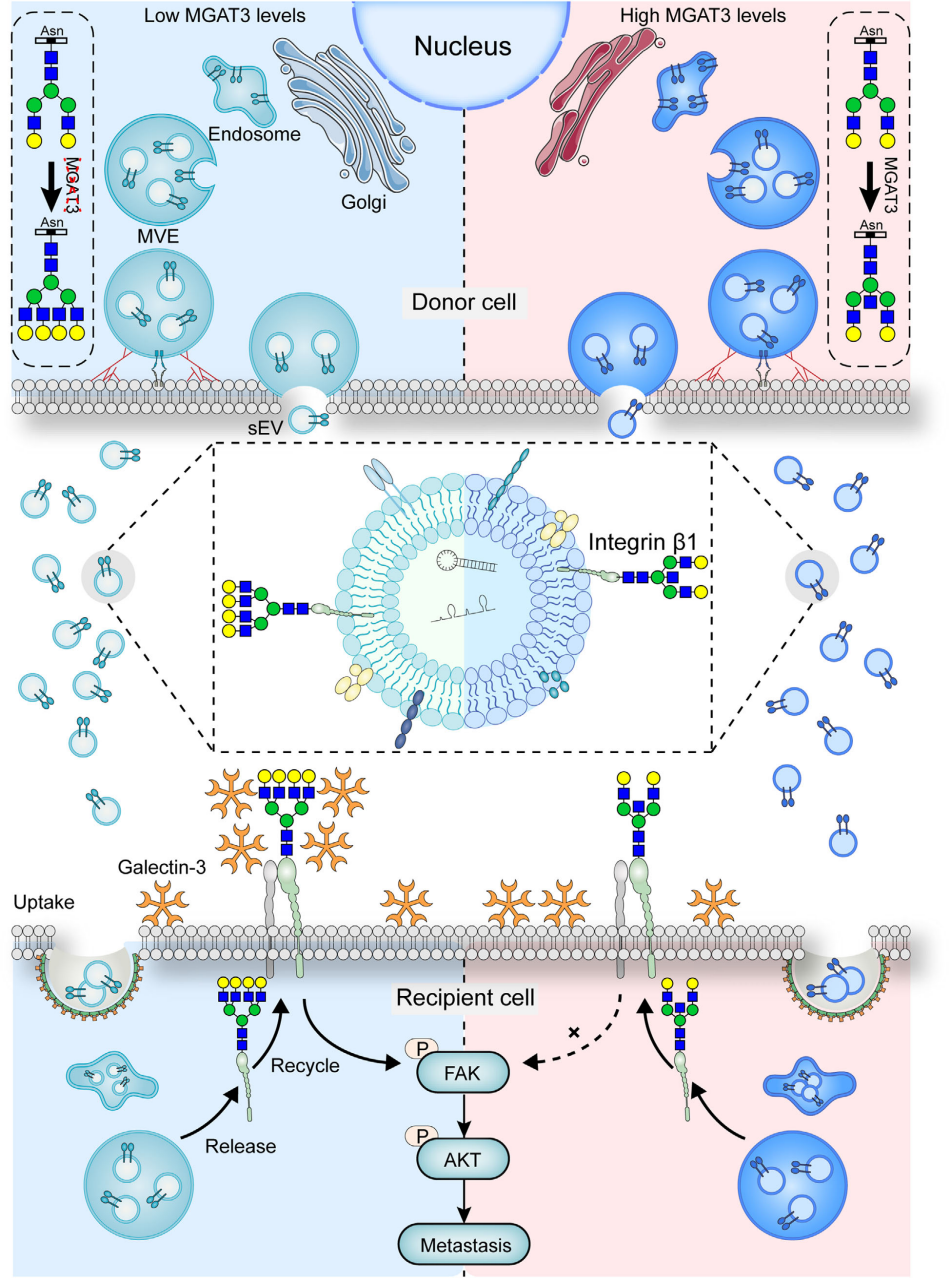
Schematic illustrating bisecting GlcNAc on sEVs suppresses metastasis of recipient cells

Glycans play important role in microvesicle protein sorting, exosome‐cell interactions, and provide promising biomarkers for various diseases. Cell‐surface intact heparan sulfate proteoglycans are necessary for internalization and functional activity of cancer cell exosomes (Christianson et al., 2013). Alteration of complex glycans could control recruitment of specific glycoprotein (e.g. EWI‐2) into exosomes/microvesicles (Liang et al., 2014). Exosomal glypican‐1 (GPC1) may serve as a potential non‐invasive diagnostic and screening tool to detect early stages of pancreatic cancer (Melo et al., 2015). Our findings clearly demonstrate that bisecting GlcNAc, a distinctive type of N‐glycosylation, is involved in BC progression, and that high level of bisecting GlcNAc suppresses metastasis of recipient cells induced by BC‐cell‐derived sEVs (Figure 8).

Many recent studies have shown that sEVs function as carriers to transport functional materials (proteins, mRNAs, microRNAs, lncRNAs) to recipient cells, and modulate recipient cell behaviour. For example, vesicular EGFR (Zhang et al., 2017), annexin A6 (ANXA6) (Keklikoglou et al., 2019) and integrin αMβ2 (Wu et al., 2020) were reported to facilitate the metastasis of recipient cells. On the other hand, several sEV cargos, were documented to suppress the cancer migration of recipient cells. For example, CD82, a potent inhibitor of cancer metastasis, were enriched in exosomes and significantly impaired cell adhesion and migration of ovarian cancer cell ES2 (Li et al., 2020). Overexpression of PTEN in donor cells were found to be enriched in exosomes, and internalization of exosomal PTEN resulted in reduced p‐AKT levels and decreased cell proliferation (Putz et al., 2012). Glycosylation has been shown to play essential roles in cancer development and progression (Pinho & Reis, 2015). sEVs, similarly to donor cells, are covered with heavy glycoconjugates. However, little is known regarding biological functions of the glycoconjugates on sEVs. We demonstrate here a suppressive function of bisecting GlcNAcylation on sEVs from BC cells. Our findings, in combination with previous observations that regulatory microRNAs and other non‐coding RNAs in sEVs mediate premetastatic properties of recipient cells and the tumour microenvironment (Rana, Malinowska, & Zöller, 2013, Zhang et al., 2015), indicate that carcinogenic properties of sEVs are suppressed by specific glycosylation, specifically bisecting GlcNAcylation.

Bisecting GlcNAc could inhibit the elongation of N‐glycans, and regulate the biosynthesis of other N‐glycan epitopes, e.g. α‐Gal N‐glycans, branching N‐glycans, and sialyl N‐glycans (Kitada et al., 2001). Aberrant expression of bisecting GlcNAc has been observed in various cancer types (Allam et al., 2014, Nyalwidhe et al., 2013). Low levels of bisecting GlcNAc facilitate the binding of adhesion molecules and receptor glycoproteins to galectin, to form a complex in cell surface, thereby enhancing cell adhesion and migration (Kariya et al., 2010). However, high levels of bisecting GlcNAc cause a reduction of galectin binding in cell surface, and suppress cell adhesion and migration (Kariya et al., 2010). Beside its functions in tumour development and progression, bisecting GlcNAc was also reported to play essential roles in certain organ growth and development (Bhattacharyya, Bhaumik, Raju, & Stanley, 2002; Kizuka et al., 2015), and in immune tolerance (Chen, Tan, Guan, & Ren, 2020).

In our study, an apparent decreased level of bisecting GlcNAc structures and its glycosyltransferase MGAT3 were detected in breast cancer cell lines, tissues and serum (Figure 1), which was accompanied by increased levels of high mannose type N‐glycans (Figure 1a), indicating an incomplete glycosylation during breast cancer progression. It is well known that glycosylation affects many physicochemical properties of glycoproteins, particularly integrins and growth factor receptors (Isaji et al., 2004; Zhao et al., 2006; Zhao et al., 2008). Using mass spectrometric analysis of glycoproteins in combination with lectin enrichment, we identified integrin β1 as a target protein with bisecting GlcNAc (Figure 4). Vesicular β1 from breast cancer cells could be transferred to recipient cells via sEVs and further promoted the migratory ability of MCF7 cells. Previous study also shown that exosomal αvβ3 transferred from tumorigenic cells to non‐tumorigenic cells can increase adhesion and migration of recipient cells (Singh et al., 2016). And integrins on tumour exosomes from organotropic human breast and pancreatic cancer cells have been reported to determine organ‐specific metastasis (Hoshino et al., 2015). However, Dennis K. Jeppesen et al. found that integrin β1 was barely detected in CD81 and CD63 positive exosomes captured by immunoaffinity from DKO‐1 and Gli36 cells, suggesting that integrin β1 is enriched in 300–900 nm microvesicles (Jeppesen et al., 2019). In our study, immunoelectron microscopy and density gradient isolation assay confirmed the present of integrin β1 on sEVs derived from MDA‐MB‐231. The discrepancy might be explained by the diversity of extracellular vesicles from different cell lines. Various modifications of bisecting GlcNAc had no effect on secretion of or β1 expression on sEVs; however, enhancement of bisecting GlcNAc on vesicular β1 significantly inhibited its binding with galectin‐3 on the cell surface, thus inhibiting FAK signaling and suppressing the cell migratory ability (Figure 6). The binding of galectin‐3 and integrins contributes to the formation of lattices that organize the proteins on the membrane surface (Fortuna‐Costa, Gomes, Kozlowski, Stelling, & Pavão, 2014) to modulate the phosphorylation of FAK and activation of downstream small GTPase proteins, resulting in the alterations in cell migration (Defilippi, Di Stefano, & Cabodi, 2006, Mitra, Hanson, & Schlaepfer, 2005). Bisecting GlcNAc can suppress the formation of branching GlcNAc structures on glycoprotein vesicular β1, block its binding with galectin‐3, and further inhibit the pro‐metastatic functions of sEVs.

Our results showed that introduction of bisecting GlcNAc on sEVs did not decrease the amount of sEV internalization to MCF7 (Figures 3e, 5g and S9B), suggesting bisecting GlcNAc has no impact on sEV uptake. Proteomics analysis showed a number of differentially regulated vesicular proteins identified in vec‐sEVs and MGAT3‐sEVs (Table S6), speculating the potential role of bisecting GlcNAc in sEVs cargo recruitment. However, certain glycoconjugates on sEV surfaces may affect the endocytosis of sEVs into recipient cells. For example, removal of N‐glycans by PNGase F or cleavage of terminal sialic acids by neuraminidase resulted in a consistent increase in uptake of glycosidase‐treated sEVs (Williams et al., 2019, Williams et al., 2018). Heparan sulfate proteoglycans (HSPGs) on cell surface function as internalizing receptors of sEVs derived from glioblastoma cell line U‐87 MG, and the uptake of sEVs could be inhibited by exogenous HS chains (Christianson et al., 2013). These data indicated the different types of glycan structures on sEVs, and their receptors on recipient cells together determine the endocytosis of sEVs.

In view of the roles of bisecting GlcNAc level documented in this study, it is important to evaluate the effects on sEVs of other proteins with bisecting GlcNAc. Modifications of glycosylation on sEVs have a clear potential to alter biological functions of sEVs, and to contribute to development of novel targets in cancer therapy.

## CONFLICT OF INTEREST

The authors report no conflict of interest.

## Supporting information



Supplementary information

Supplementary information

Supplementary information

Supplementary information

Supplementary information

Supplementary information

Supplementary information

Supplementary information
