# Supplementary material for: Bisecting GlcNAc modification diminishes the pro‐metastatic functions of small extracellular vesicles from breast cancer cells
Source: J Extracell Vesicles. 2020 Oct 30;10(1):e12005. doi: 10.1002/jev2.12005 (PMC7710122; doi:10.1002/jev2.12005)
Supplement: Supplementary file 1 — Supplementary information [file JEV2-10-e12005-s001.docx]

**Supplementary Information**

Bisecting GlcNAc modification diminishes the pro-metastatic functions of small extracellular vesicles from breast cancer cells

Zengqi Tan^1,7^, Lin Cao^1,7^, Yurong Wu^1^, Bowen Wang^1^, Zhihui Song^1^, Juhong Yang^1^, Lanming Cheng^1^, Xiaomin Yang^2,3^, Xiaoman Zhou^1^, Zhijun Dai^4,5*^, Xiang Li^6*^, Feng Guan^1*^

^1^ Joint International Research Laboratory of Glycobiology and Medicinal Chemistry, College of Life Science, Northwest University, Xi'an, 710069, P.R. China

^2^ Department of Breast Surgery, The First Affiliated Hospital of Xi'an Jiaotong University, Xi'an, 710004, P.R. China

^3^ Department of Breast Surgery, Tumor Hospital of Shaanxi Province, Xi'an, 710004, P.R. China

^4^ Department of Breast Surgery, The First Affiliated Hospital, College of Medicine, Zhejiang University, Hangzhou, 310003, P.R. China

^5^ Department of Oncology, The Second Affiliated Hospital of Xi'an Jiaotong, Xi'an, 710004, P.R. China

^6^ School of Medicine, Northwest University, Xi'an, 710069, P.R. China

^7^ Z.T. and L.C. contributed equally to this study.

***Corresponding authors:** Zhijun Dai (dzj0911@126.com), Xiang Li (xiangli@nwu.edu.cn), Feng Guan (guanfeng@nwu.edu.cn).

Keywords: Bisecting GlcNAc, small extracellular vesicles, integrin, MGAT3, breast cancer


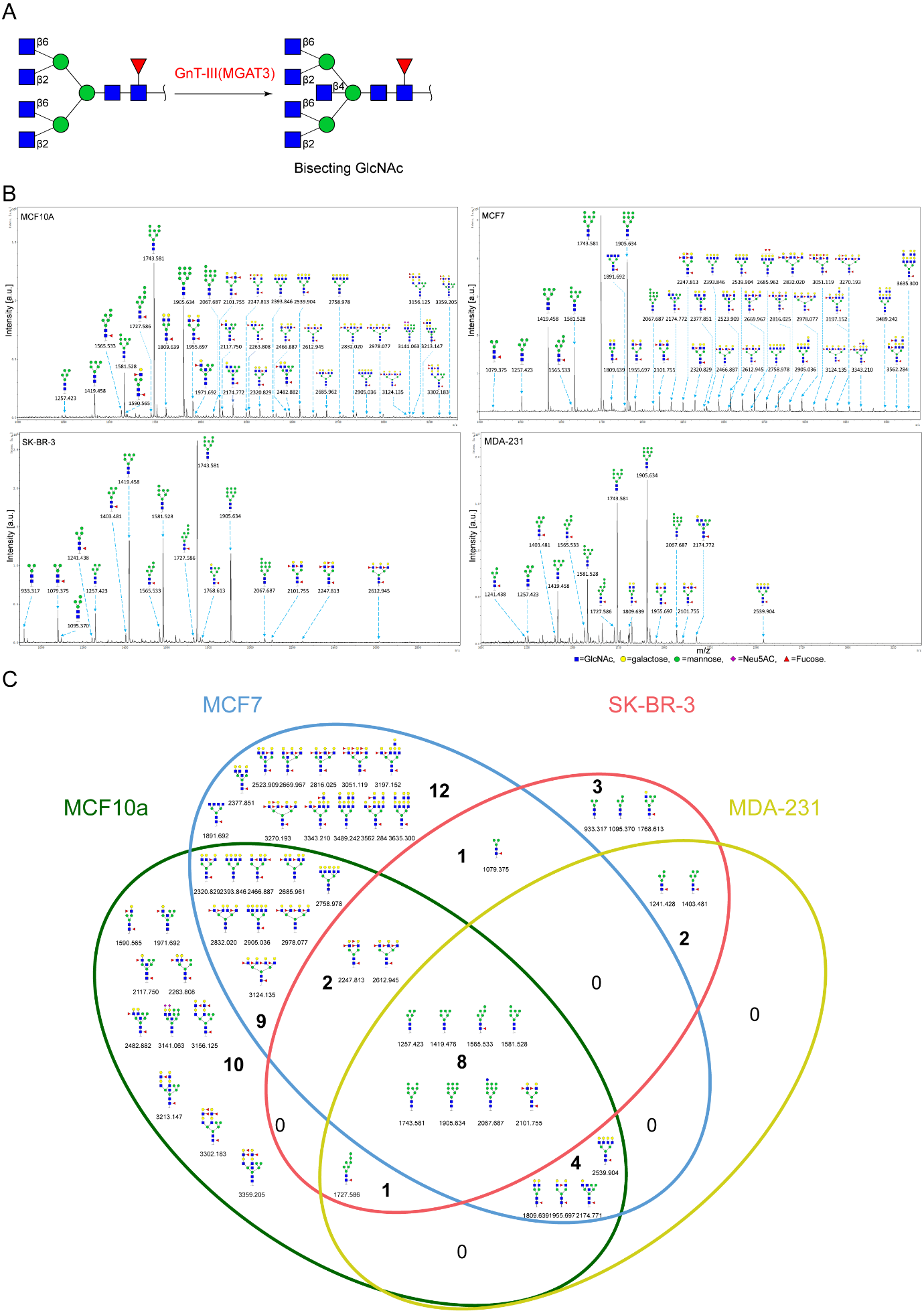


**Figure S1.** **MALDI-TOF-MS spectra of N-glycans in human breast normal epithelial and cancer cells**

**(A)**Typical structures of bisecting GlcNAc. **(B)** N-glycans from normal human epithelial MCF10A and BC cell lines MCF7, SK-RB-3, and MDA-231 were separated and desalted as described in M&M, and subjected to MALDI-TOF-MS analysis. Experiments were performed in biological triplicate, and representative N-glycan spectra are shown. Peaks (signal-to-noise ratio >5) were selected for relative proportion analysis. Detailed structures were analyzed using GlycoWorkbench software program. Proposed structures are indicated by m/z value. **(C)** Venn diagrams of numbers, structures, and m/z values of identified N-glycans from each cell line.


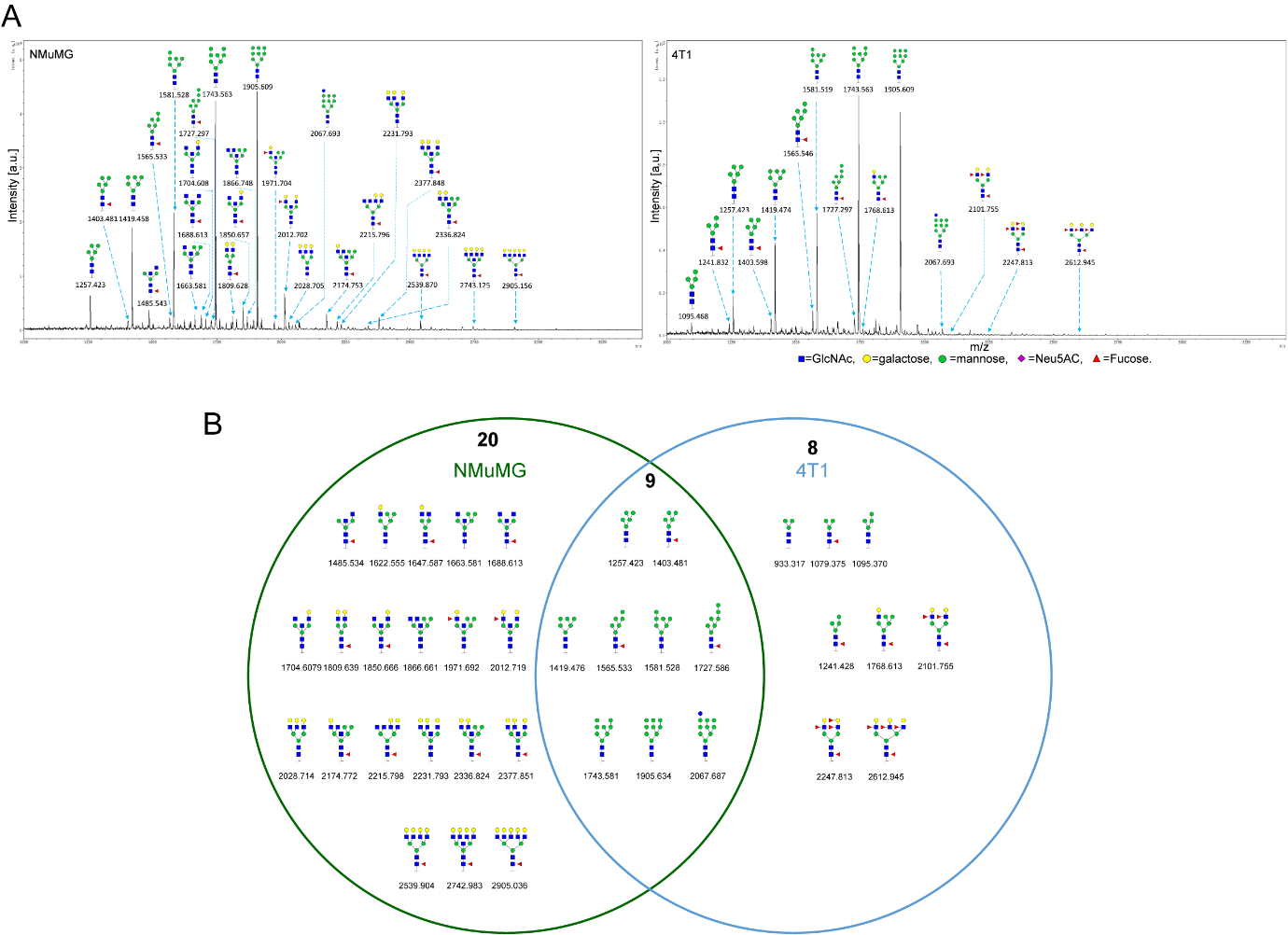


**Figure S2. MALDI-TOF-MS spectra of N-glycans in mouse breast normal epithelial and cancer cells**

**(A)** N-glycans from normal mouse mammary gland epithelial NMuMG and BC cell line 4T1 were analyzed as described in Fig. S1. **(B)** Venn diagrams of numbers, structures, and m/z values of identified N-glycans from each cell.


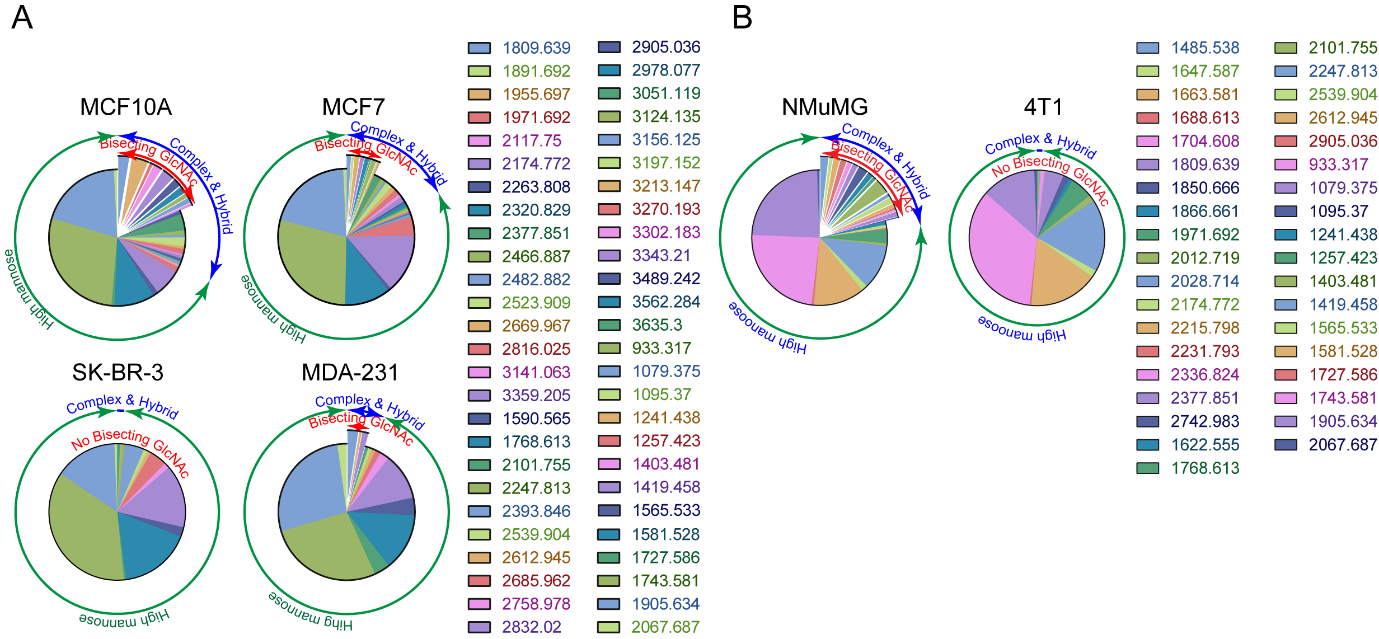


**Figure S3. Relative proportions of various types of N-glycans**

Relative proportions of N-glycans in human **(A)** and mouse **(B)** breast cells were calculated by dividing intensity of given type of N-glycan by total intensity of sample. N-glycans were classified into high-mannose types or complex & hybrid types. Relative proportions, with m/z values, are shown as pie charts. Red arrows: bisecting GlcNAc structures.


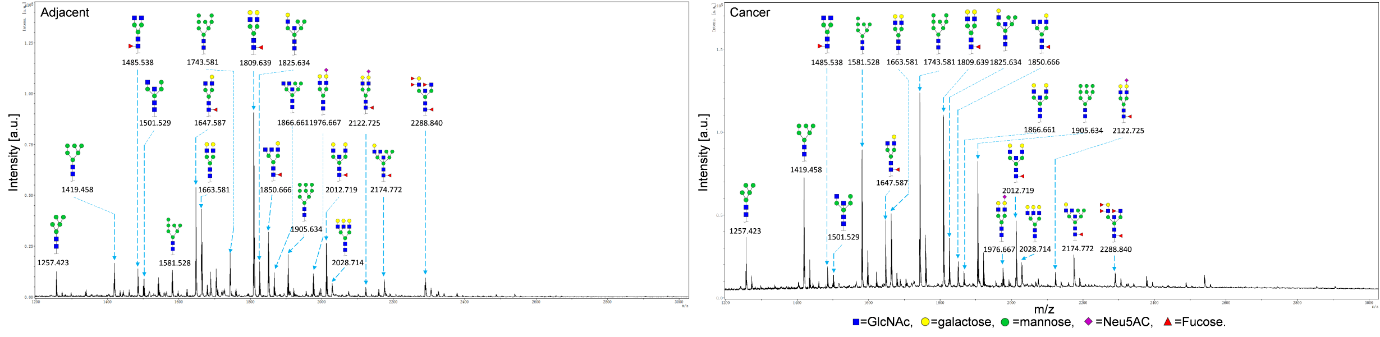


**Figure S4. MALDI-TOF-MS spectra of N-glycans in human breast tissue samples**

N-glycans from adjacent normal and matched BC tissues were analyzed as described in Fig. S1.


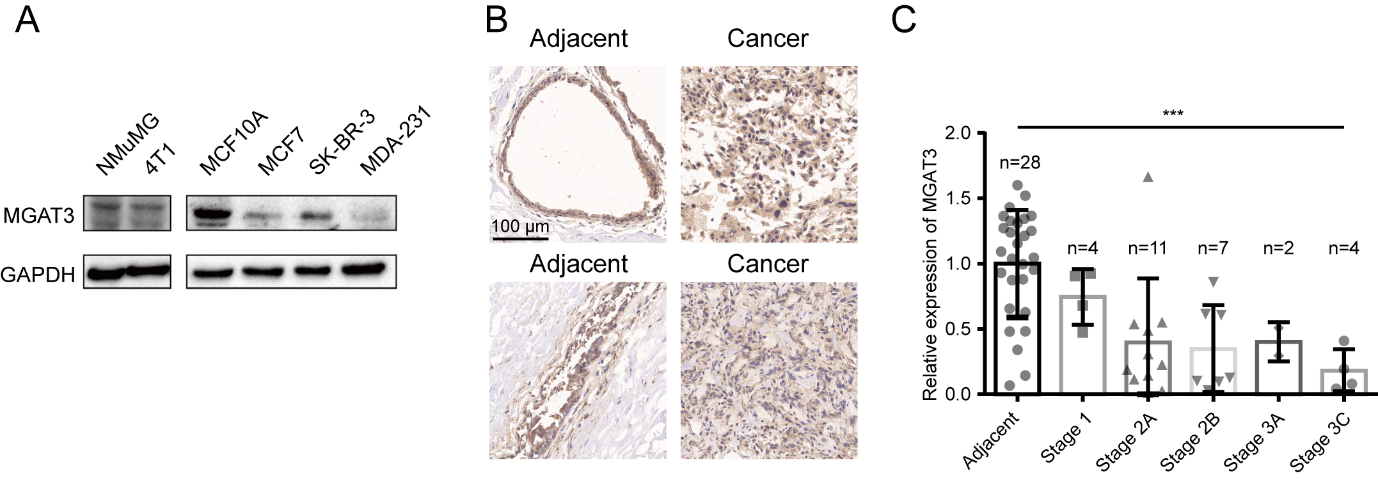


**Figure S5. Levels of bisecting GlcNAc structures and MGAT3 in breast cells and tissues**

**(A)** MGAT3 expression in human and mouse breast cells analyzed by western blotting with GAPDH as loading control. **(B)** MGAT3 expression in adjacent normal and matched BC tissues of tissue slice, by immunohistochemistry. **(C)** MGAT3 expression in various stages of BC tissue on TMAs, by immunohistochemistry.


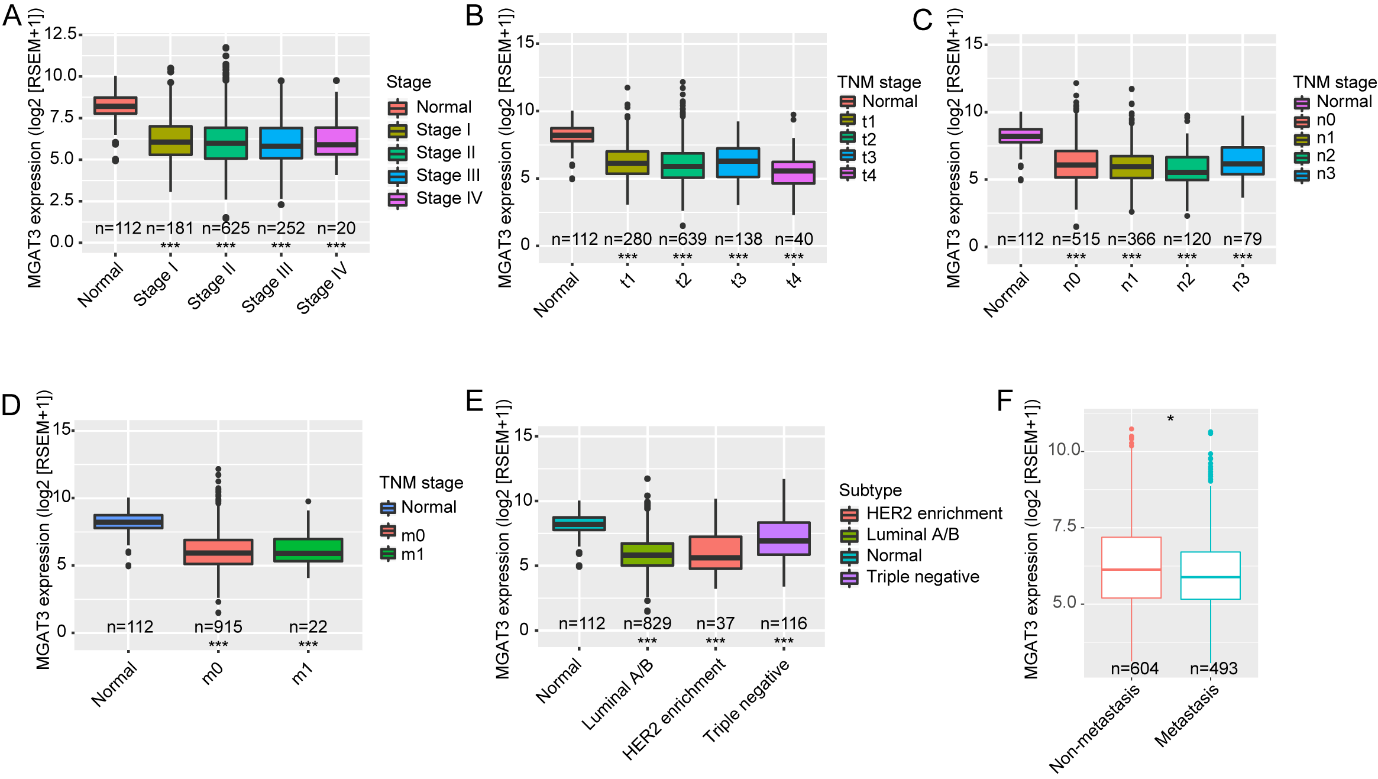


**Figure S6. MGAT3 expression at mRNA levels in various subtypes and TNM stages of BC using TCGA dataset**

**(A)** MGAT3 expression in Luminal A/B, Her2-enrichment, triple-negative BC, and adjacent normal tissues. **(B)** MGAT3 expression in various stages of BC. **(C)** MGAT3 expression in BC with various tumor sizes. **(D)** MGAT3 expression in BC with various lymph node-positive subgroups. **(E)** MGAT3 expression in BC with/without distant metastases. **(F)** mRNA expression ratio of MGAT3 in metastatic and non-metastatic breast cancer tissues in TCGA database using the RTCGAToolbox package.


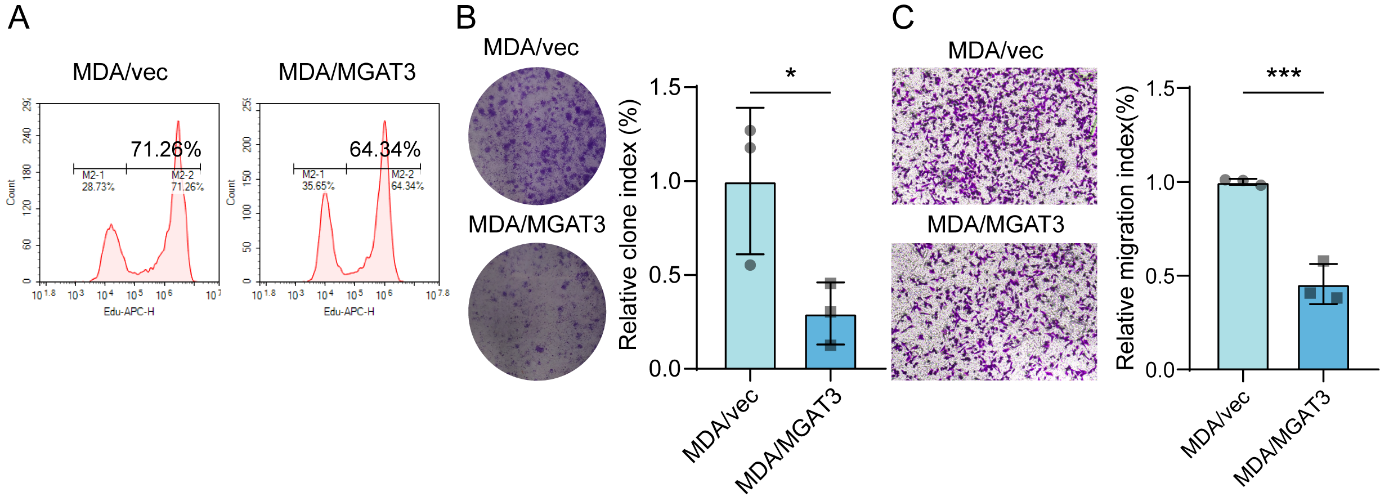


**Figure S7. Effect of bisecting GlcNAc on MDA-231 phenotypes**

**(A-C)** Proliferation **(A)**, colony formation **(B)** and migratory ability **(C)** of MDA/vec and MDA/MGAT3 cells.


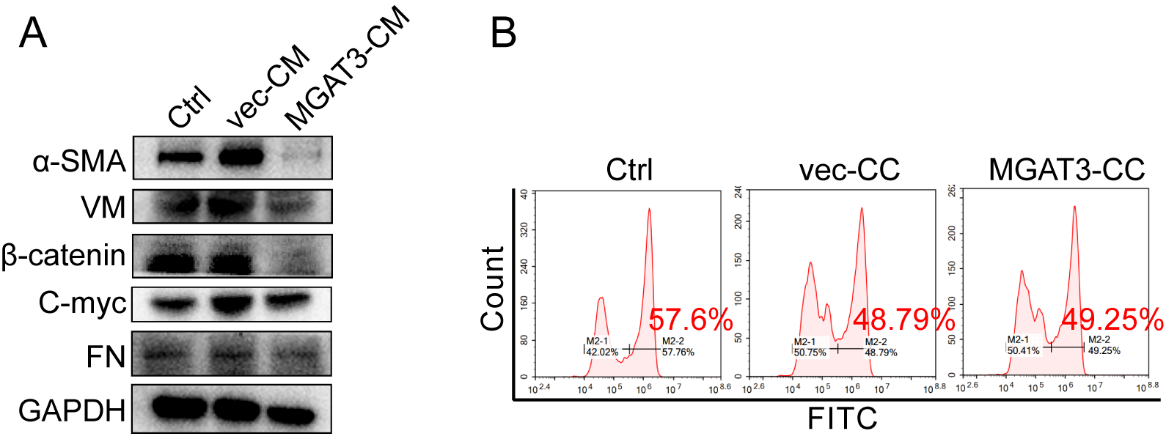


**Figure S8. Effect of conditioned medium derived from control and MDA-231/MGAT3 cells on MCF7 phenotype**

**(A)** EMT marker expression (evaluated by western blotting) of MCF7 treated with vec-CM or MGAT3-CM. **(B)** Proliferation of MCF7 co-cultured with control and MDA-231/MGAT3.


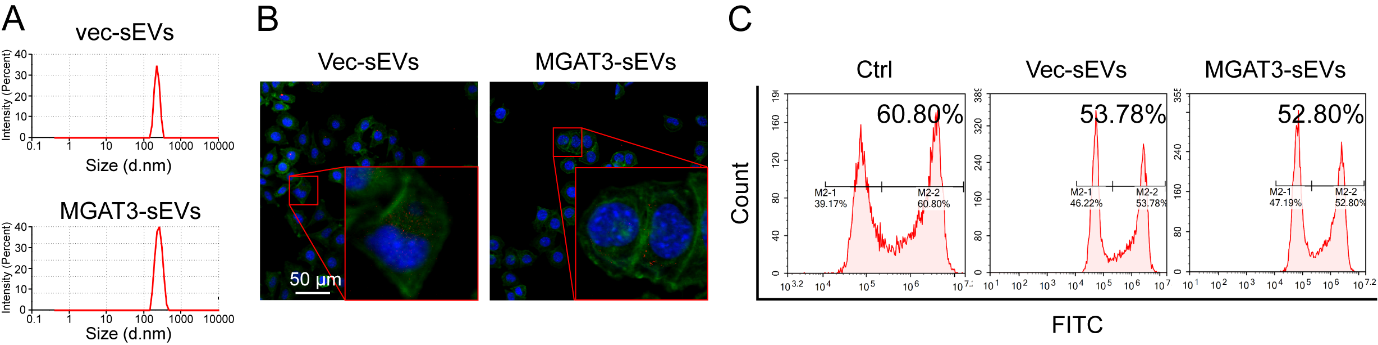


**Figure S9. Effect of bisecting GlcNAc on sEV diameter, uptake, and proliferation of recipient cells**

**(A)** Particle diameter of sEVs derived from control and MDA-231/MGAT3 cells, by Nanoparticle tracking analysis (see M&M). **(B)** Confocal analysis of sEV uptake in MCF7. Signals are indicated from a merge image of CFSE-labeled exosome (red), F-actin (green), and nucleus (blue) in MCF7 treated with vec-sEV and MGAT3-sEV. **(C)** Proliferation of MCF7 treated with vec-sEVs and MGAT3-sEVs.


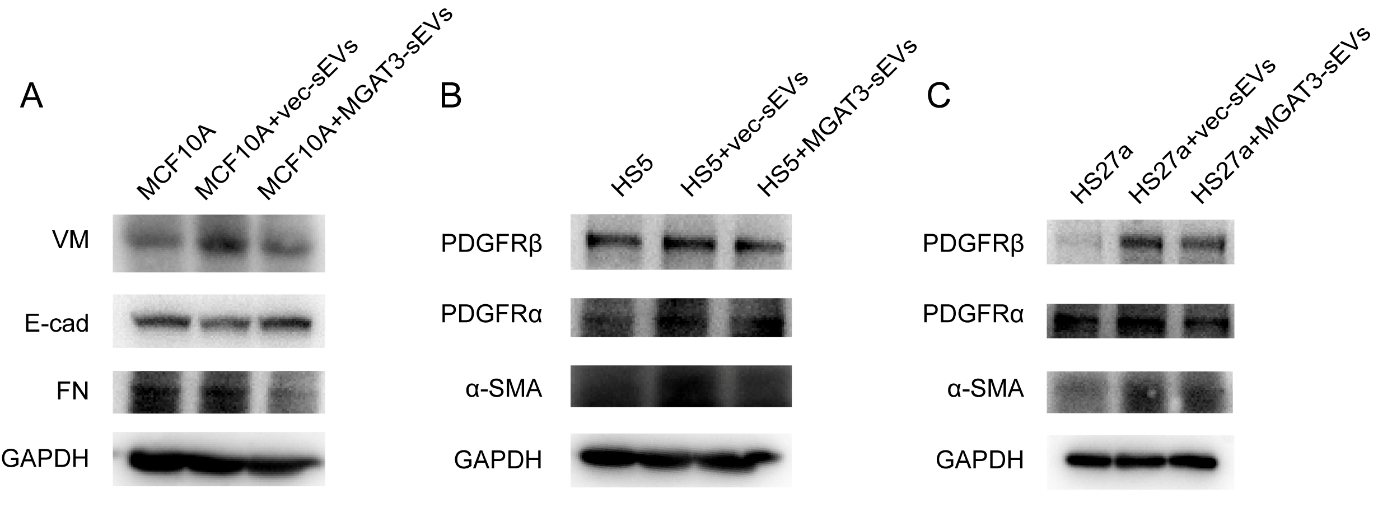


**Figure S10. Effect of vesicular bisecting GlcNAc modification on normal epithelial and stromal cells**

**(A)** Expression of EMT markers (Mesenchymal markers vimentin and fibronectin, and epithelial marker E-cadherin) in MCF10A treated with vec-sEVs and MGAT3-sEVs. **(B)** Expression of cancer-associated fibroblast (CAF) markers PDGFR α, β and SMA in HS5 treated with vec-sEVs and MGAT3-sEVs. **(C)** Expression of CAF marker in HS27a treated with vec-sEVs and MGAT3-sEVs.


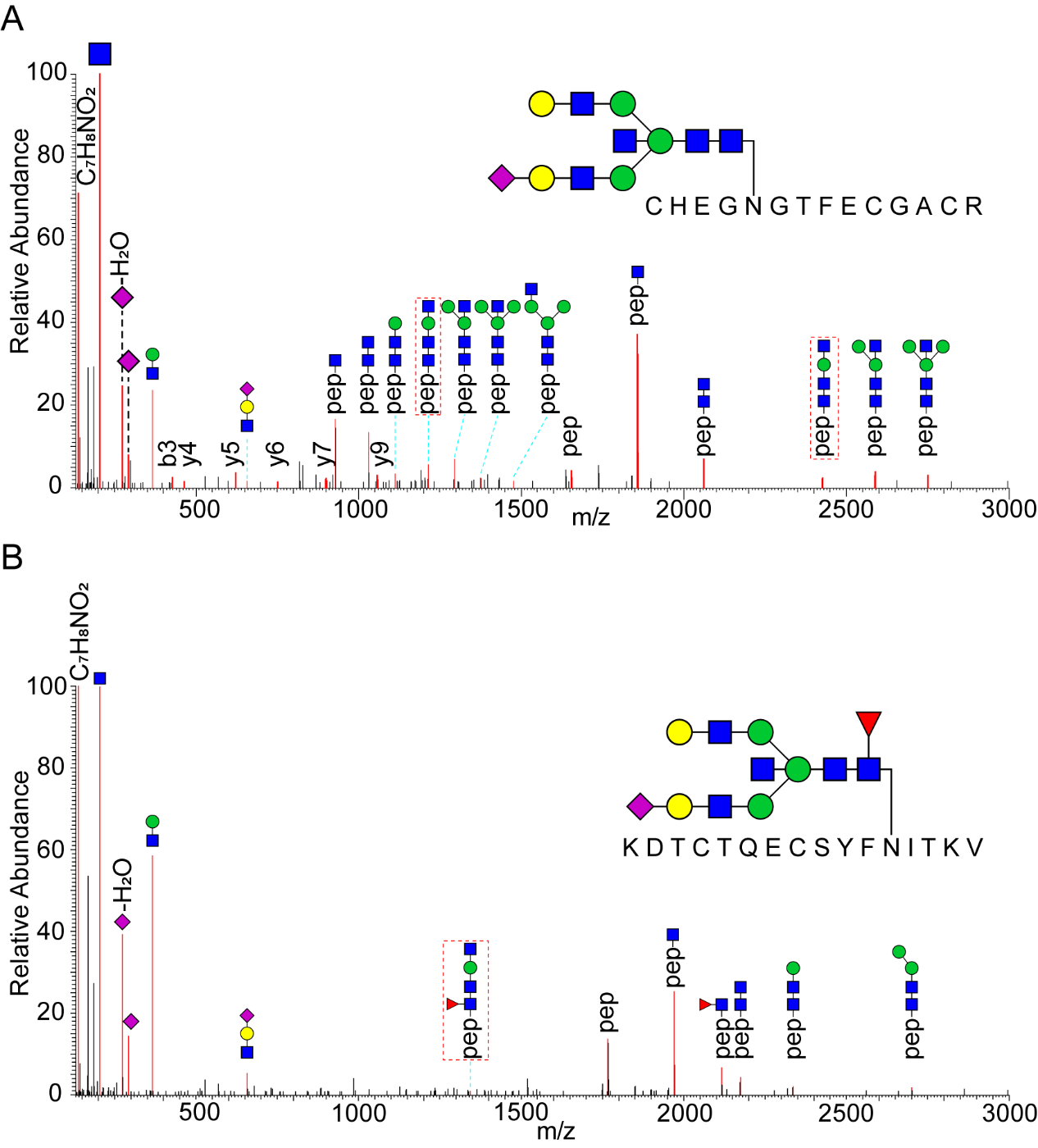


**Figure S11. Glycoproteins with bisecting GlcNAc identified in control and MDA-231/MGAT3 cells**

**(A, B)** Representative MS/MS spectrum of peptide with bisecting GlcNAc derived from β1 in MDA-231/MGAT3.


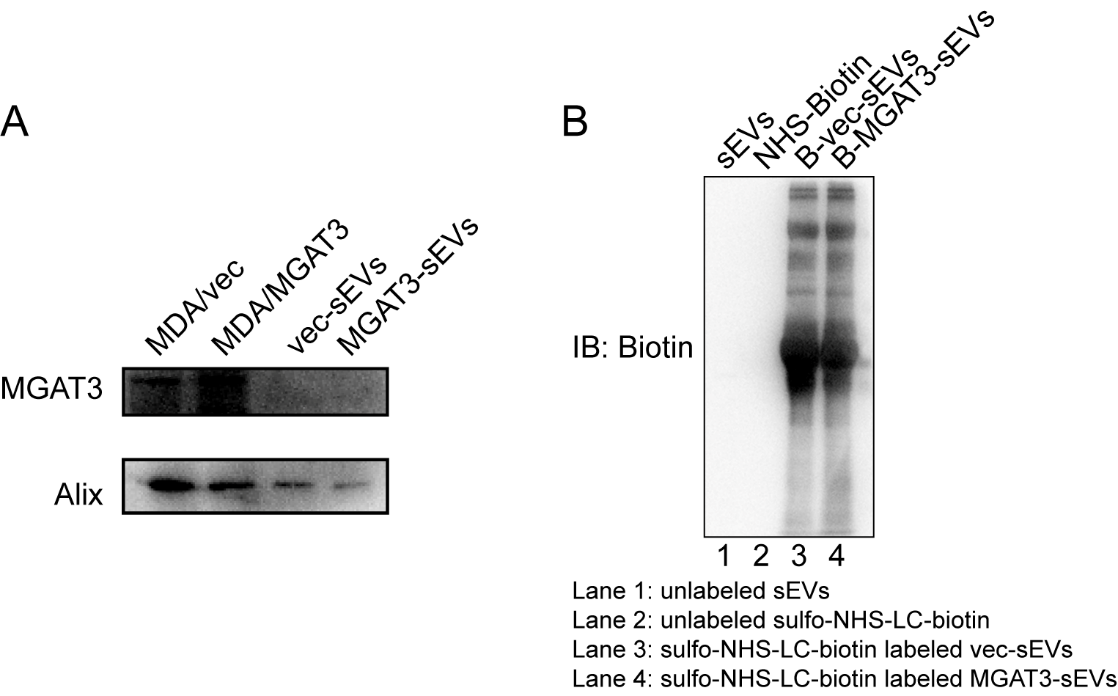


**Figure S12. Labelling of sEVs with NHS-LS-biotin**

**(A)** Levels of MGAT3 in cell lysates and sEVs of MDA/vec and MDA/MGAT3. **(B)** NHS-LC-biotin labeling of vec-sEVs and MGAT3-sEVs. Freshly collected sEVs were labeled with Sulfo-NHS-LC-Biotin, and excess biotinylation reagent was neutralized by incubation with 50 mM glycine in PBS, followed by ultracentrifugation and immunoblotting. Lane 1: unlabeled sEVs. Lane 2: unlabeled Sulfo-NHS-LC-Biotin reagent (removed after ultracentrifugation). Lane 3: Sulfo-NHS-LC-Biotin-labeled vec-sEVs. Lane 4: Sulfo-NHS-LC-Biotin-labeled MGAT3-sEVs.


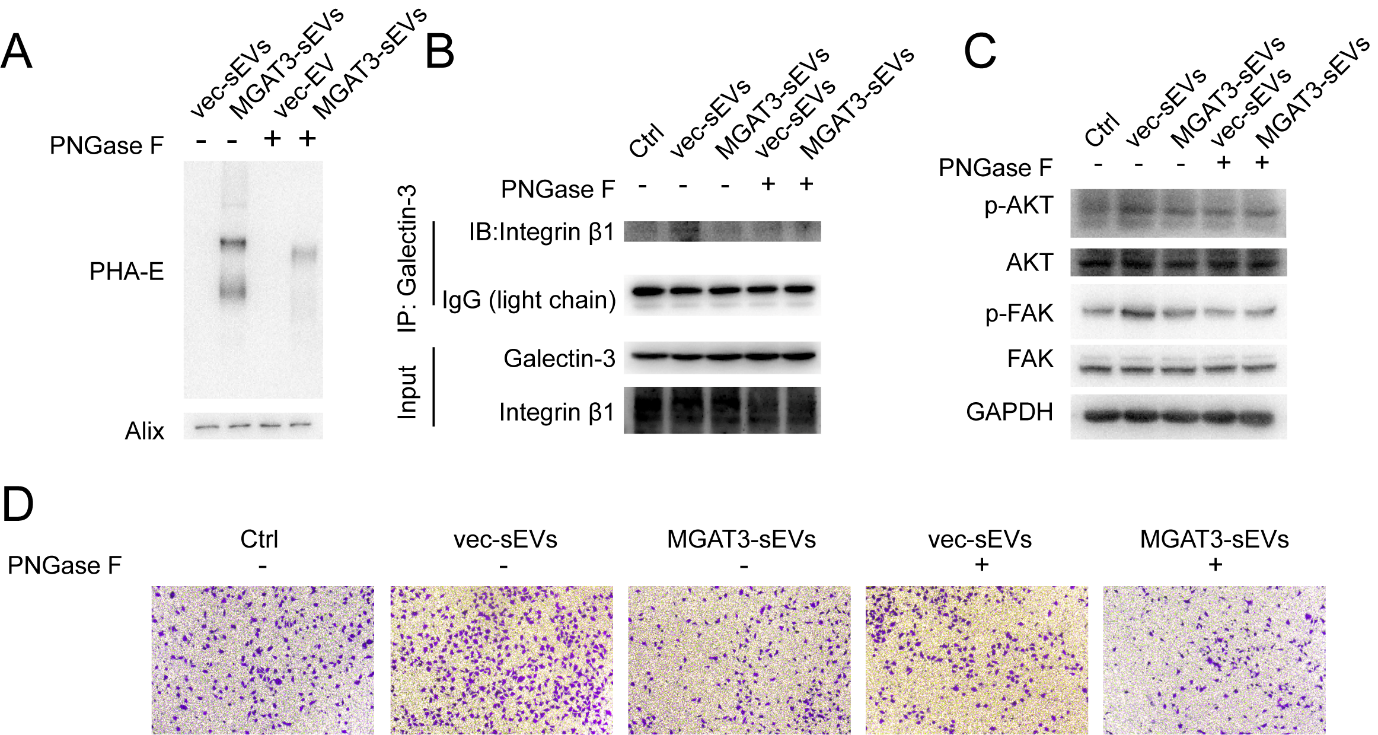


**Figure S13. Effects of removal of bisecting GlcNAc on sEVs**

**(A)** Levels of bisecting GlcNAc on sEVs after treatment with PNGase F. **(B)** Galectin-3/ β1 interaction in MCF7 treated with vec-sEVs or MGAT3-sEVs which pre-incubated with/ without PNGase F. **(C)** Activation of FAK/AKT signaling in MCF7 treated with vec-sEVs or MGAT3-sEVs which pre-incubated with/ without PNGase F. **(D)** Migratory ability of MCF7 treated with vec-sEVs or MGAT3-sEVs which pre-incubated with/ without PNGase F.


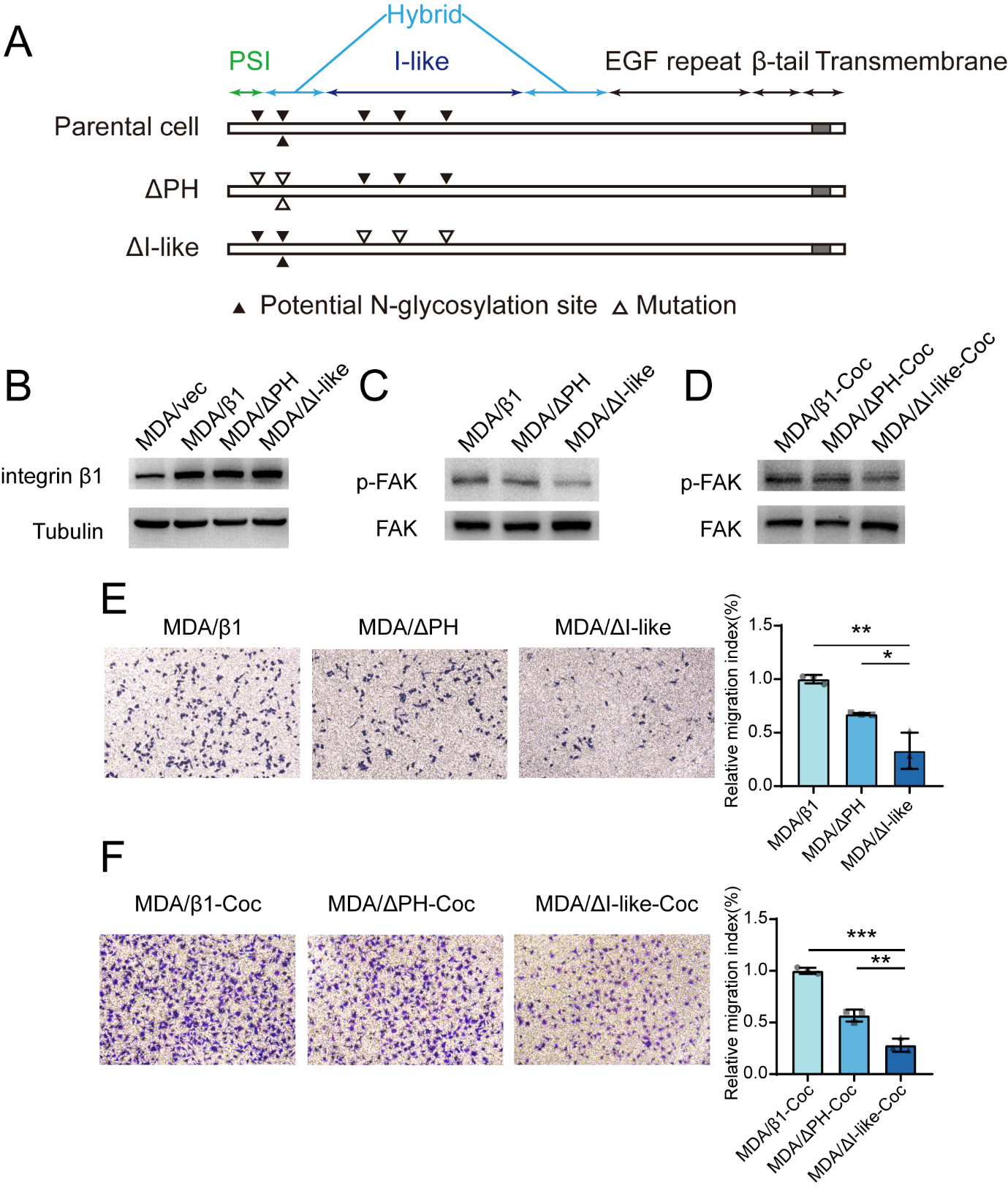


**Figure S14. Effects of glycosylation sites mutation on integrin β1**

**(A)** Schematic diagram of potential N-glycosylation sites on integrin β1 (Asn50, Asn94, Asn97, Asn212, Ans269, Asn363). N-glycosylation sites: closed triangles; point mutations: open triangels. **(B)** Intact integrin β1 (MDA/vec), PSI and upstream region of hybrid domain (MDA/ΔPH), and I-like domain (MDA/ΔI-like) mutated integrin β1 was introduced into MDA-231 cells. **(C)** Activation of FAK signaling in mutants of MDA-231 cells. **(D)** Activation of FAK signaling in MCF7 cells co-cultured with mutants of MDA-231 cells. **(E)** Migratory ability of mutants of MDA-231 cells. **(F)** Migratory ability of MCF7 cells co-cultured with mutants of MDA-231 cells.


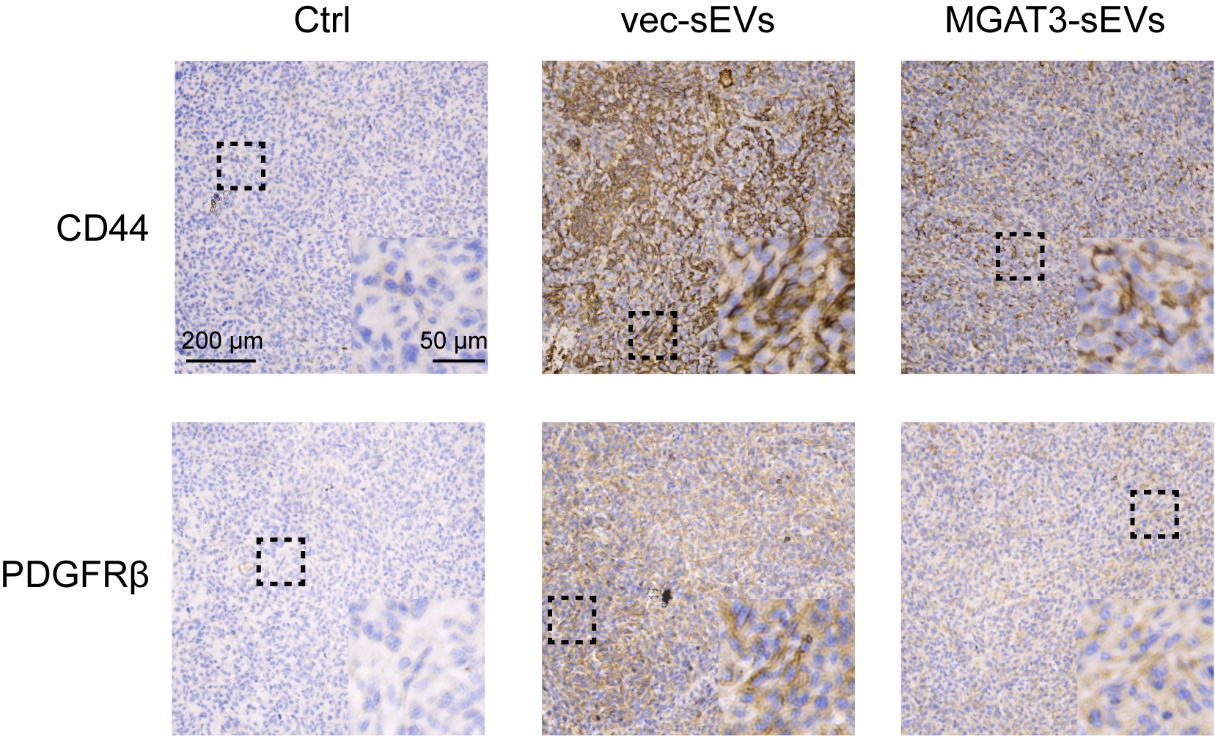


**Figure S15. Immunohistochemistry analysis of CAFs makers in PBS, vec-sEVs and MGAT3-sEVs pre-conditioned lung metastasis nodules**


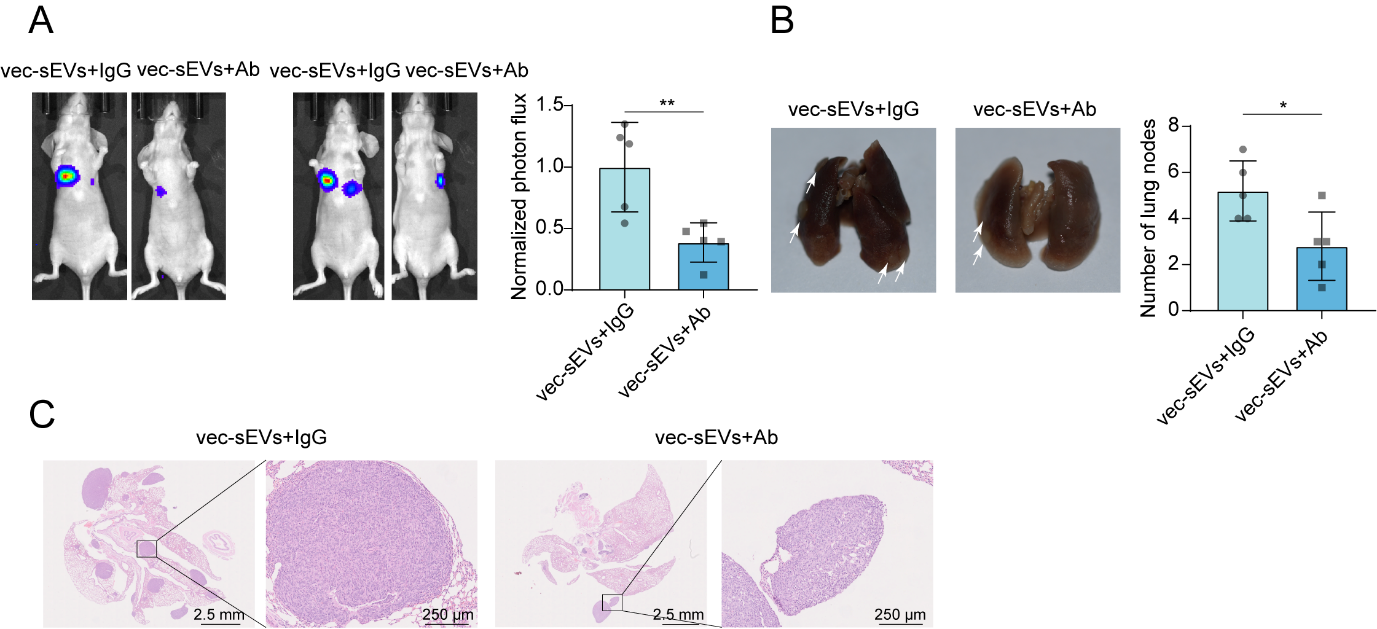


**Figure S16. Neutralized antibody against integrin β1 inhibited migratory ability of MCF7 induced by vec-sEVs**

**(A)** Luciferase activity at week 8 after injection of MCF7 pre-conditioned with vec-sEVs which pre-incubated with/ without neutralized antibody against integrin β1 (n= 5). **(B, C)** Representative photographs **(B)** and hematoxylin and eosin (H&E) staining **(C)** of lungs.
