# Supplementary material for: Bisecting GlcNAc modification diminishes the pro‐metastatic functions of small extracellular vesicles from breast cancer cells
Source: J Extracell Vesicles. 2020 Oct 30;10(1):e12005. doi: 10.1002/jev2.12005 (PMC7710122; doi:10.1002/jev2.12005)
Supplement: Supplementary file 2 — Supplementary information [file JEV2-10-e12005-s002.docx]

**Supplementary Information**

Bisecting GlcNAc modification diminishes the pro-metastatic functions of small extracellular vesicles from breast cancer cells

Zengqi Tan^1,7^, Lin Cao^1,7^, Yurong Wu^1^, Bowen Wang^1^, Zhihui Song^1^, Juhong Yang^1^, Lanming Cheng^1^, Xiaomin Yang^2,3^, Xiaoman Zhou^1^, Zhijun Dai^4,5*^, Xiang Li^6*^, Feng Guan^1*^

^1^ Joint International Research Laboratory of Glycobiology and Medicinal Chemistry, College of Life Science, Northwest University, Xi'an, 710069, P.R. China

^2^ Department of Breast Surgery, The First Affiliated Hospital of Xi'an Jiaotong University, Xi'an, 710004, P.R. China

^3^ Department of Breast Surgery, Tumor Hospital of Shaanxi Province, Xi'an, 710004, P.R. China

^4^ Department of Breast Surgery, The First Affiliated Hospital, College of Medicine, Zhejiang University, Hangzhou, 310003, P.R. China

^5^ Department of Oncology, The Second Affiliated Hospital of Xi'an Jiaotong, Xi'an, 710004, P.R. China

^6^ School of Medicine, Northwest University, Xi'an, 710069, P.R. China

^7^ Z.T. and L.C. contributed equally to this study.

***Corresponding authors:** Zhijun Dai (dzj0911@126.com), Xiang Li (xiangli@nwu.edu.cn), Feng Guan (guanfeng@nwu.edu.cn).

Keywords: Bisecting GlcNAc, small extracellular vesicles, integrin, MGAT3, breast cancer

**Materials and Methods**

**Cell lines and cell culture**

Normal mouse mammary gland epithelial cell line (NMuMG), mouse mammary carcinoma cell line (4T1), Stromal cells HS5 and HS27a were from American Type Culture Collection (Manassas, VA, USA). HS5 and HS27a, and 4T1 cells were grown in RPMI 1640 (HyClone; Logan, UT, USA) supplemented with 10% FBS (HyClone), 100 UI/mL penicillin, and 100 μg/mL streptomycin (Gibco; Carlsbad, CA, USA) at 37 °C in humidified 5% CO_2_ atmosphere. NMuMG cells were grown in DMEM containing 10 μg/mL insulin (Sigma-Aldrich; St. Louis, MO, USA), 10% FBS, 100 UI/mL penicillin, and 100 μg/mL streptomycin at 37 °C in 5% CO_2_ atmosphere.

**Analysis of N-glycans**

Total proteins (2 mg) from each cell or tissue sample were concentrated and desalted using a size-exclusion spin ultrafiltration unit (Amicon Ultra-0.5 10 KD; Millipore; Billerica, MA, USA)^1^. Proteins were denatured with 8 M urea, 10 mM dithiothreitol (DTT), and 20 mM iodoacetamide (IAM) (Sigma-Aldrich), and further digested with PNGase F (New England BioLabs; Ipswich, MA, USA) overnight at 37 °C. Released N-glycans were collected, lyophilized, and desalted using HyperSep Hypercarb (Thermo Fisher) solid phase extraction (SPE) cartridge. Desalted N-glycans were characterized by MALDI-TOF/TOF-MS (UltrafleXtreme; Bruker Daltonics; Bremen, Germany). Lyophilized N-glycans and 20 mg/mL 2,5-dihydroxybenzoic acid (DHB) were spotted onto an MTP AnchorChip sample target and air-dried. Measurements were taken in positive-ion mode, and m/z data were analyzed and annotated using GlycoWorkbench software program (http://code.google.com/p/glycoworkbench/)^2^.

**Glycosylation sites mutation**

The cDNA of integrin β1 was amplified by PCR from reverse-transcribed product of MDA-231 RNA and then inserted into pMD 18-T vector (Takara) to obtain the recombinant plasmid PMD-ITGB1. The asparagine of the glycosylation sites on the PSI and upstream region of the hybrid domain (termed ΔPH), and I-like domain of integrin β1 (ΔI-like) were replaced by an arginine in MDA-231 cells by overlap extension PCR. Mutagenesis of ΔPH and ΔI-like were made using overlap extension PCR with PMD-ITGB1 as template. The oligonucleotides used to introduce mutations are listed below and the nucleotides substituted are underlined. PCR products were inserted into vector pLVX-AcGFP1-N1 (Takara). Stable mutants were established as described in M&M/ "Stable transfection of MGAT3".

ITGB1-full length-forward 5’- CCGGAATTCatgaatttacaaccaattttctggattggact

ΔPH-N50D-reverse 5’- tgttgaatctgtgcaccacccacaatttgg (nt 127-156)

ΔPH-N50D-forward 5’- tggtgcacagattcaacatttttacaggaagg (nt 139-170)

ΔPH-N94&77D-reverse 5’- gctacggtcggttacatctttatttttctttat (nt 265-297)

ΔPH-N94&77D-forward 5’- aataaagatgtaaccgaccgtagcaaaggaac (nt 274-305)

ΔI-like-N212D-forward 5’- ctggtgcagtcctgttcacttgtgc (nt 620-650)

ΔI-like-N212D-reverse 5’- tgaacaggactgcaccagcccatt (nt 627-650)

ΔI-like-N269D-forward 5’- tgtaacatccctccagccaatcagtg (nt 788-813)

ΔI-like-N269D-reverse 5’- tggagggatgttacacggctgc (nt 799-820)

ΔI-like-N363D-forward 5’- cattgctagaatctgcagataatgttcc (nt 1072-1099)

ΔI-like-N363D-reverse 5’- tatctgcagattctagcaatgtaattcagtt (nt 1079-1109)

ITGB1-full length-reverse 5’- GCTCTAGAtcattttccctcatacttcggattgac

1. Yang G.*, et al.* Selective isolation and analysis of glycoprotein fractions and their glycomes from hepatocellular carcinoma sera. *Proteomics* **13**, 1481-1498 (2013).

2. Ceroni A., Maass K., Geyer H., Geyer R., Dell A. & Haslam S. M. GlycoWorkbench: a tool for the computer-assisted annotation of mass spectra of glycans. *Journal of Proteome Research* **7**, 1650-1659 (2008).
