# Supplementary material for: Bisecting GlcNAc modification diminishes the pro‐metastatic functions of small extracellular vesicles from breast cancer cells
Source: J Extracell Vesicles. 2020 Oct 30;10(1):e12005. doi: 10.1002/jev2.12005 (PMC7710122; doi:10.1002/jev2.12005)
Supplement: Supplementary file 7 — Supplementary information [file JEV2-10-e12005-s007.docx]

|  | Low | High |
| --- | --- | --- |
| Age (mean) | 50.6 | 58.2 |
| Lymph node (LN) invasion | | |
| Patients with positive LN | 88.9% | 62.5% |
| Number of LN (mean) | 2.56 | 1.5 |
| Pathological tumor size (mm) (mean) | 1.83 | 1.8 |
| Hormonal receptor | | |
| Positive | 88.9% | 100% |
| Pathological type | | |
| Ductual | 100% | 100% |
| Lobular | 0% | 11.1% |
